# Notch signaling and efficacy of PD-1/PD-L1 blockade in relapsed small cell lung cancer

**DOI:** 10.1038/s41467-021-24164-y

**Published:** 2021-06-23

**Authors:** Nitin Roper, Moises J. Velez, Alberto Chiappori, Yoo Sun Kim, Jun S. Wei, Sivasish Sindiri, Nobuyuki Takahashi, Deborah Mulford, Suresh Kumar, Kris Ylaya, Christopher Trindade, Irena Manukyan, Anna-Leigh Brown, Jane B. Trepel, Jung-Min Lee, Stephen Hewitt, Javed Khan, Anish Thomas

**Affiliations:** 1 Developmental Therapeutics Branch, Center for Cancer Research, NCI, NIH, Bethesda, MD USA; 2 Department of Pathology and Internal Medicine, University of Rochester, Rochester, NY USA; 3 Moffitt Cancer Center, Tampa, FL USA; 4 Genetics Branch, Center for Cancer Research, NCI, NIH, Bethesda, MD USA; 5 Laboratory of Pathology, Center for Cancer Research, NCI, NIH, Bethesda, MD USA; 6 National Center for Biotechnology Information, NIH, NLM, Bethesda, MD USA; 7 Women’s Malignancies Branch, Center for Cancer Research, NCI, NIH, Bethesda, MD USA

**Keywords:** Small-cell lung cancer, Tumour immunology, Cancer genomics, Small-cell lung cancer

## Abstract

Immune checkpoint blockade (ICB) benefits only a small subset of patients with small cell lung cancer (SCLC), yet the mechanisms driving benefit are poorly understood. To identify predictors of clinical benefit to ICB, we performed immunogenomic profiling of tumor samples from patients with relapsed SCLC. Tumors of patients who derive clinical benefit from ICB exhibit cytotoxic T-cell infiltration, high expression of antigen processing and presentation machinery (APM) genes, and low neuroendocrine (NE) differentiation. However, elevated Notch signaling, which positively correlates with low NE differentiation, most significantly predicts clinical benefit to ICB. Activation of Notch signaling in a NE human SCLC cell line induces a low NE phenotype, marked by increased expression of APM genes, demonstrating a mechanistic link between Notch activation, low NE differentiation and increased intrinsic tumor immunity. Our findings suggest Notch signaling as a determinant of response to ICB in SCLC.

## Abstract (web summary)

Immune checkpoint blockade (ICB) benefits only a small subset of patients with small cell lung cancer (SCLC) and the mechanisms driving benefit are poorly understood. Here, the authors show that elevated Notch signaling predicts clinical benefit in ICB in relapsed SCLC.

## Introduction

Small cell lung cancer (SCLC) is a highly aggressive neuroendocrine (NE) cancer that accounts for ~15% of all lung cancer, with an annual incidence of more than 34,000 in the United States alone. Treatment of SCLC has historically consisted of chemotherapy with platinum and etoposide, which leads to responses in most patients, but resistance quickly develops^1^.

SCLC is characterized by the loss of function of p53 and RB1^2^ and high tumor mutational burden (TMB), which suggests that these tumors could be immunogenic and respond to immune checkpoint blockade (ICB). However, the benefit from ICB in an unselected SCLC population is modest. For first-line treatment of SCLC, the addition of atezolizumab, an anti-programmed death ligand 1 (PD-L1) antibody, to carboplatin and etoposide in the phase 3 IMpower133 trial^3^ significantly improved median survival from 10.3 to 12.3 months; however, only 12.6% of patients remained progression-free at 1 year. Similarly, in the phase 3 CASPIAN trial^4^, the addition of durvalumab, another anti-PD-L1 antibody, to chemotherapy significantly improved median survival from 10.3 to 13 months; however, only 18.0% of patients remained progression-free at 1 year. Response to single-agent anti-PD-1/PD-L1 therapy in second- or third-line treatment of SCLC occurs in only 10–20% of patients^5,6^. Nonetheless, 3–8% of patients with relapsed SCLC experience durable responses, suggesting there is a small subset of SCLC patients who derive significant clinical benefit (CB)^5–8^.

Despite increasing evidence that biologically distinct subsets of SCLC exist^9^, reliable predictors of treatment efficacy have not yet been developed. Unlike some other cancers such as non-small-cell lung cancer, tumor PD-L1 expression is infrequent in SCLC^10^, and its utility as a predictive biomarker of ICB responses is controversial^3,11,12^. Improved ICB response rates were suggested in relapsed SCLC with high TMB in one retrospective study^13^, but in the IMpower133 trial^3^, treatment benefit was observed regardless of pre-specified TMB thresholds.

A major barrier to discovery of mechanisms underlying ICB response in SCLC is the lack of access to tumor tissue for research. Few patients undergo surgery as the disease is usually extensively disseminated by the time it comes to medical attention^14^. Biopsies at relapse are typically outside of standard care. Indeed, SCLC is not included in The Cancer Genome Atlas (TCGA) that contains over 11,000 tumors from more than 30 different tumor types.

Given the significant heterogeneity in SCLC^9^, ascertaining the subsets of SCLC that have CB to ICB is critical. In the present study, we evaluate the immunogenomic features associated with clinical outcomes in patient with relapsed SCLC to gain insight into the underlying mechanisms of ICB response. Using prospective and retrospective anti-PD-1/PD-L1 antibody-treated SCLC cohorts, we find an association between high expression of Notch pathway genes and CB to ICB. Moreover, across four additional SCLC cohorts, tumors with high expression of Notch pathway genes are associated with low NE differentiation which, in turn, predicts elevated expression of antigen processing and presenting machinery (APM) and cytotoxic infiltrating immune cell genes. In vitro, activation of Notch signaling induces a low NE phenotype that is characterized by increased expression of APM genes. Our findings suggest Notch signaling as a determinant of CB to anti-PD-1/PD-L1 therapies in relapsed SCLC.

## Results

### Study design and clinical results

**Fig. 1:**
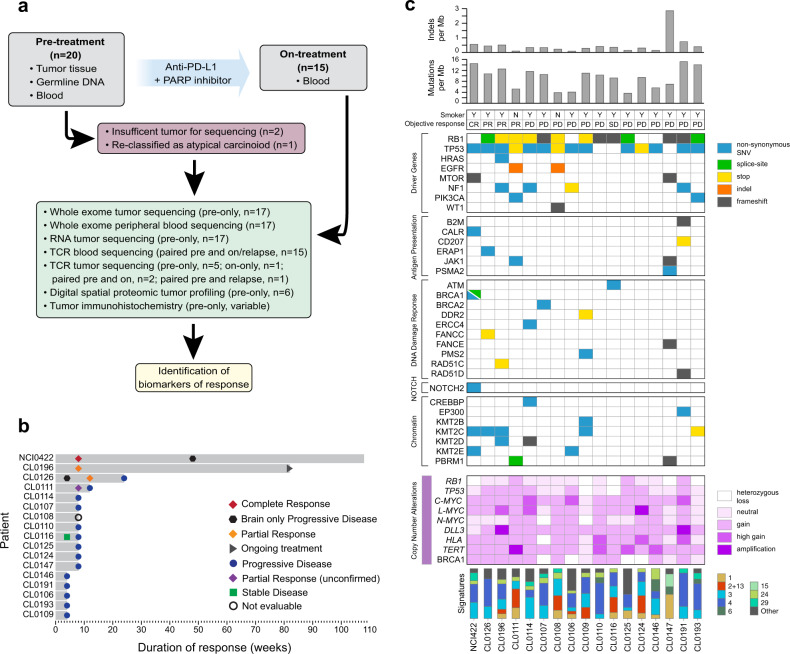
Study design, clinical characteristics, and whole exome sequencing summary results of the relapsed small cell lung cancer discovery cohort. **a** Schematic representation of the clinical trial of combination immune checkpoint blockade (PD-L1 inhibitor plus PARP inhibitor) and sample analysis. Tumor samples were collected prior to treatment and analyzed using whole exome sequencing, RNA sequencing, digital spatial profiling, and immunohistochemistry. T-cell receptor sequencing was performed on pre- and on-treatment blood samples, and on select tumors, where available. **b** Swimmer plot indicating response and length of time on therapy for individual patients. Timing of responses are also indicated. **c** Heatmap summarizing mutation burden, mutational signatures, somatic variants and copy number alterations derived from whole exome sequencing of patient tumors. Smoking status and best response for each patient are also shown. Source data are provided as a Source Data file.

Our discovery cohort was derived from a prospective clinical trial of 20 previously treated patients with SCLC who received durvalumab, an anti-PD-L1 antibody administered every 4 weeks and olaparib, a poly (ADP-ribose) polymerase (PARP) inhibitor administered twice daily^15^ (Fig. 1a). Baseline clinical characteristics of the patients have been previously described^15^. Four patients derived CB from the treatment. Patient NCI0422 had a confirmed complete response (CR) by Response Evaluation Criteria in Solid Tumors (RECIST 1.1) after 8 weeks on treatment, and the response was maintained for 1 year (Fig. 1b). Thereafter, the patient had relapse in the cerebellum, which was resected. The systemic CR was maintained for another year (Fig. 1b), when the patient died of complications related to tumor involvement of the brain and spinal cord. Patient CL0196 had small cell carcinoma that transformed from an Epidermal Growth Factor Receptor (EGFR) mutant lung adenocarcinoma following anti-EGFR therapy and had a confirmed partial response (PR) after 8 weeks on treatment with an ongoing response at data cutoff (Fig. 1b). Patient CL0111 had a PR at 8 weeks after treatment but had tumor progression soon after (unconfirmed PR) (Fig. 1b). Patient CL0126 had brain-only progressive disease (PD) shortly after starting therapy but had a systemic PR that lasted for 6 months (Fig. 1b). The remaining patients had no clinical benefit (NCB) and developed PD 4–8 weeks after starting treatment (Fig. 1b and Supplementary Data 1).

In addition to clinical activity, one key objective of this study was to evaluate predictive biomarkers of CB to the ICB combination. The trial design included a mandatory pre-treatment biopsy to specifically interrogate this question. Tumor biopsies and blood were obtained before treatment, and blood was collected at multiple on-treatment timepoints. Representative slides of tumor samples were reviewed and SCLC was confirmed prior to patients receiving therapy. Re-review of pathology after trial completion revealed one tumor with features suggestive of large cell NE carcinoma (LCNEC; CL0108), one with focal adenocarcinoma component (CL0111) and one with atypical features of SCLC (prominent nucleoli not exceeding 10% of the population; NCI0422) (Supplementary Fig. 1). Review of an additional biopsy from NCI0422 prior to first-line treatment revealed SCLC with typical features (Supplementary Fig. 2E). One patient, CL0150, was re-classified as an atypical carcinoid^16^ and therefore this patient was removed from our analysis. Detailed clinical, histopathology data and available H&E images of the tumors included in the final analyses are provided in Supplementary Data 1 and Supplementary Fig. 1.

We performed wholeexome sequencing (WES) and RNA sequencing of pre-treatment tumors, WES of DNA from peripheral blood mononuclear cells, and digital spatial proteomic profiling and immunohistochemistry (IHC) of pre-treatment tumors to identify genomic, transcriptomic and proteomic correlates of CB to ICB (Fig. 1a). Two patients did not have sufficient tumor content for sequencing (Fig. 1a). Tumor purity was high across all sequenced samples (median 0.85) (Supplementary Data 1). T-cell receptor (TCR) sequencing was performed on pre- and on/relapse treatment blood from 15 patients as well as on 2 paired pre- and on-treatment tumors, 1 paired pre-treatment and at relapse tumors, 5 pre-treatment only tumors, and 1 on-treatment only tumor (Fig. 1a and Supplementary Data 1).

### Genomic alterations do not correlate with clinical benefit to combination immune checkpoint blockade in relapsed SCLC

We first sought to identify correlates of CB to combination ICB from WES (Supplementary Data 2). Consistent with previous reports^2^, we found *RB1* or *TP53* genomic alterations (somatic variants or copy-number alterations) in all cases [100% (*n* = 17/17)] (Fig. 1c). Amplifications in *MYC* family genes occurred in 29% (*n* = 5/17) of patients with both CB and NCB (Fig. 1c). One patient with CB and one patient with NCB had sensitizing mutations in *EGFR* (CL0111 and CL0108, respectively) (Fig. 1c). Other mutated genes including *HRAS*, *MTOR*, *NF1, PIK3CA*, and *WT1* were distributed across the cohort (Fig. 1c). Genomic alterations in APM pathways were uncommon. However, one patient, CL0107 (NCB) displayed homozygous loss of *B2M* (Fig. 1c), which has been associated with acquired resistance to ICB^17^. Nonsynonymous tumor mutation burden was similar between patients with CB and those with NCB (Fig. 1c).

Given previous reports suggesting alterations in DNA-damage response (DDR) genes predicting CB to ICB^18,19^, we assessed somatic variants in DDR genes in our cohort. Loss of function variants (frameshift, stop, indel) in DDR pathways occurred in 50% (*n* = 2/4) of CB patients compared to 23% (*n* = 3/13) of NCB patients (Fig. 1c). Patient NCI0422 (CB) had a pathogenic somatic *BRCA1* E1512Q variant (Fig. 1c). However, there was no evidence of *BRCA1* loss of heterozygosity (Fig. 1c) nor *BRCA1/2* germline defects that would suggest biallelic inactivation of *BRCA1*. While we cannot rule out the possibility that the PARP inhibitor olaparib contributed to NCI0422’s CR, given *BRCA1* mutations are rare in SCLC^2^ and SCLC is not a BRCA-associated cancer type^20^, it is unlikely to have accounted for the CR to the ICB combination. Our cohort had no loss of function variants in *NOTCH1-4*, which occur frequently in SCLC^2^. NCI0422 had a nonsynonymous mutation in the N-terminal EGF-like repeats domain of *NOTCH2* (Fig. 1c). However, the ligand-binding site of Notch receptor is frequently mutated across cancer types; therefore, the impact of such mutations on Notch signaling is unclear^21^. Mutations in epigenetic pathways were frequent across both CB and NCB patients; most were nonsynonymous, and loss of function mutations were infrequent (Fig. 1c). Lastly, mutational signatures associated with smoking (signature 4), homologous recombination (signature 3) and APOBEC mutagenesis (signatures 2 + 13) were common across the cohort and were not enriched among CB or NCB patients (Fig. 1c). One patient, CL0147 (NCB), displayed evidence of mismatch repair defects based on mutational signature profiling (Fig. 1c).

### Immune and Notch signaling gene sets are associated with clinical benefit to combination immune checkpoint blockade in relapsed SCLC

**Fig. 2:**
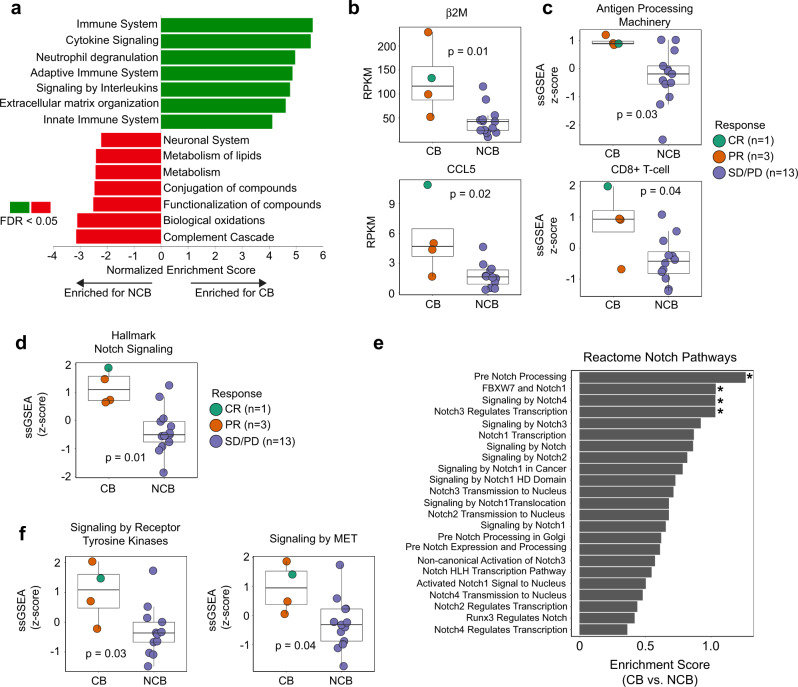
Notch signaling and immune gene sets are associated with clinical benefit to combination immune checkpoint blockade in the relapsed small cell lung cancer discovery cohort. **a** Bar plot of Reactome gene sets enriched in tumors of patients with clinical benefit (CB) and no clinical benefit (NCB) to immune checkpoint blockade (ICB) based on differential gene expression analysis. **b** Expression of immune-related genes, **c** ssGSEA immune gene set scores, and **d** ssGSEA Hallmark Notch signaling gene set score in tumors of patients with and without clinical benefit following ICB. **e** Enrichment of Reactome gene sets related to Notch signaling in patients with CB to ICB. Asterisks denote significantly enriched gene sets (unadjusted *p* < 0.05) **f** ssGSEA scores of Reactome gene sets related to receptor tyrosine kinase and MET activation in tumors of patients with and without clinical benefit following ICB. Each circle represents a tumor from a given patient; green: complete response; orange: partial response; blue: stable or progressive disease. Statistical significance was tested with a two-tailed Mann–Whitney *U*-test. Boxplots represent the median, 25th and 75th percentiles and the vertical bars span the 5th to the 95th percentiles. Source data are provided as a Source Data file.

Given the lack of genomic alterations associated with CB to combination ICB, we next assessed gene expression profiles using RNA-seq (Supplementary Data 3). Differential gene expression revealed strong enrichment of immune-related pathways among patients with CB compared to NCB (FDR < 0.05) (Fig. 2a and Supplementary Data 4). Expression of APM genes (*β2M*) and tumor immune microenvironment genes (chemokine *CCL5*) were also higher in patients with CB compared to NCB (unadjusted *p* = 0.01 and 0.02, respectively) (Fig. 2b). Consistent with gene expression, digital spatial proteomic profiling^22^ of B and T-cell immune markers revealed higher counts of CD3 and CD45 in CB patients compared to NCB patients, with the largest increases evident in patient NCI0422 (CB), although these results were not statistically significant (Supplementary Fig. 2A and Supplementary Data 5).

We next sought to determine if tumors from patients with CB harbored any common biological or molecular characteristics. Patients with CB had less enrichment of neuronal and metabolism pathways compared with patients with NCB (FDR < 0.05) (Fig. 2a). NE pathway genes such as dopa decarboxylase were significantly decreased among patients with CB compared to NCB (unadjusted *p* = 0.02) (Supplementary Fig. 2B). To further define NE differentiation in our cohort, we employed a previously described signature consisting of genes associated with high and low NE differentiation such as *ASCL1* and *YAP1,* respectively^23^. Across the cohort, there was a strong negative correlation between the level of NE differentiation and the expression of immune-related genes (*R* = −0.62, *p* = 0.008) (Supplementary Fig. 2D). Three of the four CB patients (75%) displayed transcriptomic evidence of low NE differentiation including patient NCI0422 (CB), who displayed a strong, low NE phenotype; however, NE differentiation was not significantly associated with CB to ICB (Supplementary Fig. 2C). By IHC, all tumors expressed synaptophysin except CL0114 (NCB) and NCI0422 (CB) both of which had focal/weak expression (Supplementary Data 1). An additional biopsy from NCI0422 showed only focal expression of both synaptophysin and insulinoma-associated protein 1 (INSM1), a validated and sensitive marker of NE differentiation^24^ (Supplementary Fig. 2E). NCI0422 (CB) also showed high intra-tumoral CD8+ T cell infiltration (Supplementary Fig. 2F). In contrast, there was diffuse expression of synaptophysin and no intra-tumoral CD8^+^ T-cell infiltration in CL0106 (NCB) (Supplementary Fig. 2F).

To further elucidate pathways that may be associated with CB to ICB, we performed a single sample gene set enrichment analysis (ssGSEA) using CIBERSORT^25^ and Hallmark gene sets^26^. CIBERSORT ssGSEA demonstrated increased APM and CD8^+^ T-cell gene sets (unadjusted *p* = 0.03 and 0.04, respectively) in patients with CB compared to NCB consistent with results from our differential expression analysis (Fig. 2c). Hallmark ssGSEA analysis revealed Notch signaling, a major driver of NE differentiation^27^, as the most significantly enriched gene set among patients with CB (unadjusted *p* = 0.01) (Fig. 2d and Supplementary Data 6). Using a broader set of Reactome gene sets, we found positive enrichment of the majority of Notch-related gene sets among patients with CB (Fig. 2e and Supplementary Data 7). Additional significantly enriched gene sets among patients with CB to ICB included receptor tyrosine kinase signaling and MET activation (Fig. 2f and Supplementary Data 7), both of which have been associated with immune infiltrated tumors with mesenchymal features^28^. Altogether, these transcriptomic and proteomic data suggest that CB from combination ICB in relapsed SCLC is significantly associated with an immune-rich tumor microenvironment and high expression of Notch signaling genes.

### Alterations in peripheral blood T-cell repertoire and T-cell clonotype expansion are associated with clinical benefit to combination immune checkpoint blockade

We next examined changes in the peripheral blood T-cell repertoire before and on-treatment using deep TCR sequencing. Among patients with NCB and those with CB with PRs, there was no change in clonality and Jensen–Shannon Divergence (Supplementary Fig. 3A, C and Supplementary Data 8) and a minor shift in richness (Supplementary Fig. 3B and Supplementary Data 8). Remarkably, however, patient NCI0422 (CB), who had a CR, had changes in all diversity metrics compared to patients with NCB and CB with PR that were consistently observed across multiple timepoints (Supplementary Fig. 3A–C and Supplementary Data 8). We validated the observed changes in diversity metrics in patient NCI0422 using an alternative RNA-based TCR-seq method (Supplementary Fig. 3A–C and Supplementary Data 8).

We next tracked individual peripheral blood clonotypes in each patient to further understand changes in the T-cell repertoire, particularly in patient NCI0422. Very few clonotypes expanded (defined as fold change greater than 3 between on-treatment and pre-treatment with a minimum of 10 reads) among CB patients with PR and patients with NCB (range 0–152). In contrast, patient NCI0422 had a large expansion of clonotypes (range 702–1945) (Supplementary Fig. 3D and Supplementary Data 8) demonstrating both the presence and durability of T-cell expansion in response to combination ICB. To further understand how peripheral blood T-cell expansion may have impacted tumor regression in patient NCI0422, we compared peripheral blood TCR-seq to TCR-seq of the pre-treatment tumor and of the cerebellar tumor at relapse. Eleven percent of expanded peripheral blood clones were present in the pre-treatment tumor whereas only 2% were present in the relapsed CNS tumor (Supplementary Fig. 3E and Supplementary Data 8). Moreover, six T-cell clones expanded in at least 80% of peripheral blood samples were present in the pre-treatment tumor but absent in the CNS relapsed tumor (Supplementary Fig. 3F and Supplementary Data 8). Together, these results suggest the CR exhibited by patient NCI0422 to combination ICB was likely mediated by the expansion of pre-existing intra-tumoral T cells.

Lastly, we analyzed additional TCR-seq on eight pre-treatment tumors and three on-treatment tumors to assess differences between patients with and without CB. The number of unique clonotypes in the pre-treatment tumors of patient CL0111 (CB) and patient NCI0422 (CB) were higher than in the tumors of NCB patients (Supplementary Fig. 3G and Supplementary Data 8). There was minimal increase in the number of unique clonotypes in paired pre-post-treatment tumors from NCB patients with available TCR-seq (Supplementary Fig. 3H and Supplementary Data 8). Moreover, the number of clonotypes in post-treatment tumors from NCB patients was less than the number of clonotypes in the post-treatment tumor from patient CL0126 (CB) (Supplementary Fig. 3H and Supplementary Data 8). These results support our RNA-seq analyses, demonstrating the association between tumor T-cell infiltration and CB to combination ICB.

### Validation of the association of immune and Notch signaling gene sets with clinical benefit in relapsed SCLC patients treated with immune checkpoint blockade

**Fig. 3:**
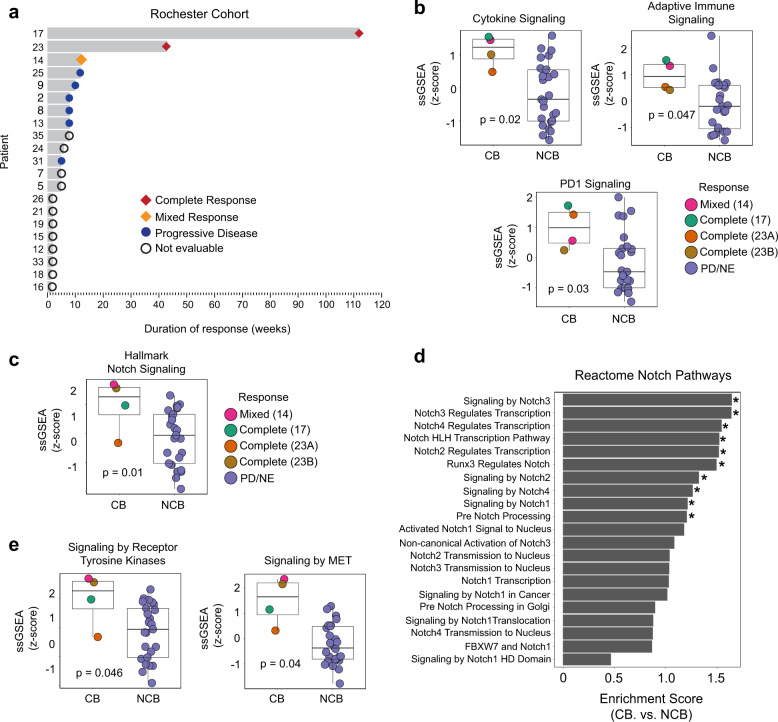
Validation of the association between Notch signaling and immune gene sets and clinical benefit to immune checkpoint blockade in relapsed small cell lung cancer. **a** Swimmer plot indicating response and length of time on therapy for individual patients in the Rochester validation cohort. Patients with clinical benefit (CB) are those with complete or mixed response and patients with no clinical benefit (NCB) are those with progressive or not evaluable disease. **b** ssGSEA Reactome immune gene set scores and **c** ssGSEA Hallmark Notch signaling gene set score in tumors of patients with and without clinical benefit to ICB. Each circle represents a tumor from a given patient. **d** Enrichment of Reactome gene sets related to Notch signaling in patients with clinical benefit to ICB. Asterisks denote significantly enriched gene sets (unadjusted *p* < 0.05). **e** ssGSEA scores of Reactome gene sets related to receptor tyrosine kinase and MET activation in tumors of patients with and without clinical benefit following ICB. Statistical significance was tested with a two-tailed Mann–Whitney *U* test. Boxplots represent the median, 25th and 75th percentiles and the vertical bars span the 5th to the 95th percentiles. Timing of responses are not shown for individual patients in swimmer plot. PD progressive disease, NE not evaluable disease. Source data are provided as a Source Data file.

We next recruited an independent cohort of patients with relapsed SCLC treated with ICB to validate findings from our discovery cohort. The Rochester cohort consisted of 36 patients treated with nivolumab, an anti-PD1 antibody, of whom 22 had sufficient material for RNA sequencing. One patient was later determined to have been treated for squamous cell carcinoma, and was excluded from further analysis (Supplementary Fig. 4). Transcriptomes of a total of 29 tumors were sequenced, including 7 patients who had 2 or more tumors sequenced (Supplementary Data 9 and 10). Available H&E images of tumors in this cohort are shown in Supplementary Fig. 5. Among the sequenced cases, three patients had CB: patients 17 and 23 had complete and durable responses (112 and 42 weeks, respectively) and patient 14 had a mixed response with significant shrinkage in one tumor but growth in another (full clinical details available in Supplementary Data 9); the remaining patients had either disease progression or unevaluable disease due to rapid progression (median treatment duration 4 weeks) (Fig. 3a and Supplementary Data 9). Cytokine, adaptive immune system, and PD-1 signaling were among the significantly enriched gene sets (unadjusted *p* = 0.02, 0.047 and 0.03, respectively) in tumors of patients with CB (Fig. 3b and Supplementary Data 11), similar to the NCI discovery cohort. Tumors of patients with CB displayed significantly higher ssGSEA scores of the Hallmark Notch signaling gene set than patients with NCB (unadjusted *p* = 0.01) (Fig. 3c and Supplementary Data 12). Moreover, similar to the discovery cohort, there was both an enrichment of Reactome Notch gene sets and significant associations between Reactome gene sets related to receptor tyrosine kinases and MET activation among patients with CB to ICB (Fig. 3d, e and Supplementary Data 13). In regard to NE differentiation by RNA-seq, tumors of patients with CB had low NE scores (14, 17, 23A/B), but there were also a number of tumors from patients with NCB with low NE scores (2, 7, 8, 12B, 15A/B, 16B, 26, 31A/B, 35) (Supplementary Data 14). Tumors from patients with NCB expressed moderate to strong levels of synaptophysin by IHC except 12B (Supplementary Data 9). Tumors of patients with CB showed weak (patient 14) and negative (patient 17) expression of synaptophysin except patient 23 who had tumors with more than 50% (A) and 80% (B) staining (Supplementary Data 9). INSM1 was positive among all patients with NCB but was only available in only 1 of 4 tumors from patients with CB (tumor 23B which was positive for INSM1) (Supplementary Data 9).

### Notch signaling is the most significant predictor of clinical benefit to immune checkpoint blockade across both relapsed SCLC cohorts

**Fig. 4:**
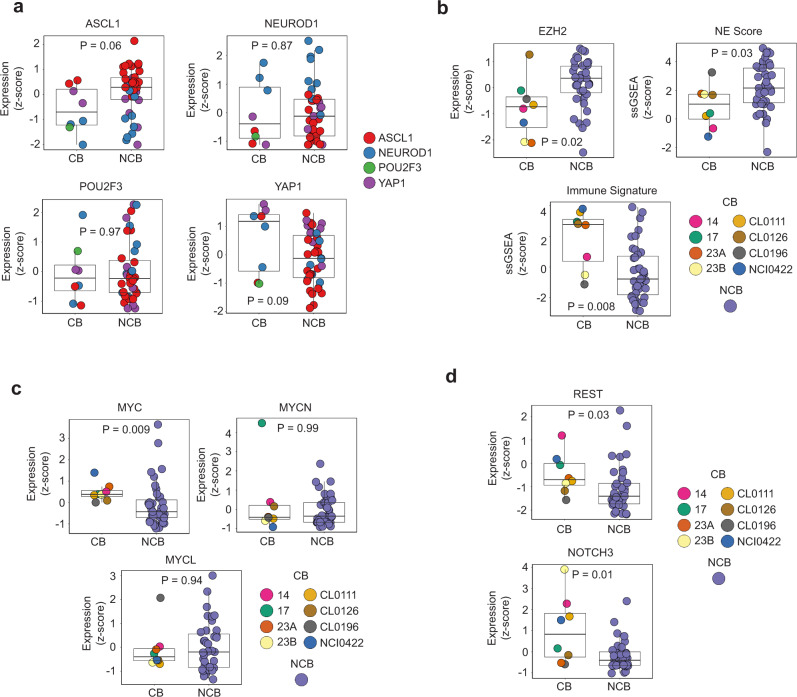
Variables associated with clinical benefit to immune checkpoint blockade across both relapsed small cell lung cancer cohorts. Association between clinical benefit to ICB and expression of **a** transcriptional subtype genes, **b** *EZH2* and ssGSEA NE differentiation and immune signature scores, **c** MYC family members (*MYC*, *MYCN* and *MYCL*) and **d** established Notch signaling target genes. Statistical significance was tested with a two-tailed Mann–Whitney *U* test. Boxplots represent the median, 25th and 75th percentiles and the vertical bars span the 5th to the 95th percentiles. Source data are provided as a Source Data file.

We next assessed the recently proposed transcriptional subtypes of SCLC^9^ based on expression of lineage transcription factors *ASCL1*, *NEUROD1*, *POU2F3*, and *YAP1* among tumors in the ICB-treated cohorts. Patients with CB in the NCI discovery cohort had tumors classified as *NEUROD1*^high^ (CL0111 and CL0126), *POU2F3*^high^ (CL0196), and *ASCL1*^high^ (NCI0422) (Supplementary Fig. 6 and Supplementary Data 14). Within the Rochester cohort, patients with CB had tumors of the *YAP1*^high^ (14 and 23B), *ASCL1*^high^ (23A) and *NEUROD1*^high^ (17) subtypes (Supplementary Fig. 6 and Supplementary Data 14). While tumors of patients who derived CB were not enriched within a specific transcriptional subtype (Table 1), we reasoned that expression of *ASCL1* and *YAP1*, which are strongly negatively and positively regulated by Notch signaling, respectively^2^, may be lower in patients with CB to ICB. Combining the ICB-treated cohorts, we found that expression of *ASCL1* was indeed lower among patients with CB to ICB, although statistical significance was not reached (unadjusted *p* = 0.06) (Fig. 4a). Conversely, *YAP1* expression was higher among patients with CB to ICB but did not reach statistical significance (unadjusted *p* = 0.09) (Fig. 4a). Although Notch signaling is known to repress *NEUROD1* expression^29,30^, there was no significant association between *NEUROD1* expression and CB to ICB (unadjusted *p* = 0.87) (Fig. 4a). *POU2F3* expression was also not significantly associated with CB to ICB (unadjusted *p* = 0.97) (Fig. 4a).

**Table 1:** Association between transcriptional subtypes and clinical benefit to immune checkpoint blockade across relapsed SCLC cohorts.

Transcriptional subtype	Clinical benefit (# of tumors)	No clinical benefit (# of tumors)	*p* value^a^
ASCL1	2	22	0.11
NEUROD1	3	9	0.40
POU2F3	1	0	0.17
YAP1	2	8	0.78

^a^Statistical significance calculated using the two-tailed chi-squared test.

In addition to lineage transcription factors, SCLC tumors are classified by expression of *MYC* family of transcription factors (*MYC*, *MYCL* and *MYCN*)^31^, NE status^23^ and expression of epigenetic genes such as EZH2^32,33^. Across both cohorts, immune signature was significantly higher (unadjusted *p* = 0.008, respectively) while NE score and EZH2 expression were significantly lower (unadjusted *p* = 0.03 and *p* = 0.02, respectively) among tumors of patients with CB compared to NCB (Fig. 4b). *MYC* was strongly associated with CB (unadjusted *p* < 0.001), whereas *MYCL* and *MYCN* were not (unadjusted *p* = 0.99 and *p* = 0.94, respectively) (Fig. 4c). To ascertain how these additional factors may predict CB to ICB, we performed a multivariable analysis. This analysis demonstrated that Notch signaling was the only significant predictor of CB to ICB across both cohorts (FDR = 5.9 × 10^−4^) (Table 2). In addition, we found significantly higher expression of two well-validated Notch signaling target genes, *REST* (unadjusted p = 0.03) and *NOTCH3* (unadjusted *p* = 0.01) in patients with CB to ICB (Fig. 4d). There were no significant differences in expression of Notch ligands (*DLL1*, *DLL3*, *JAG1*, *DLK1*) between patients with and without CB (Supplementary Data 14).

**Table 2:** Notch signaling gene set is the most significant predictor of clinical benefit to immune checkpoint blockade across relapsed SCLC cohorts.

Variable	Estimate^a^	*t* value^a^	*p* value^a^	FDR^b^
Hallmark Notch signaling	0.25	4.31	9.8 × 10^−4^	5.9 × 10^−4^
Immune signature	0.13	2.06	0.047	0.14
NE score	−0.07	−1.82	0.08	0.16
MYC expression	−0.04	−0.83	0.41	0.62
EZH2 expression	−0.03	−0.56	0.58	0.62

Outcome dependent variable = clinical benefit to immune checkpoint blockade.

^a^Estimates, *t* and *p* values calculated using multivariable logistic regression.

^b^False discovery rate was calculated using the Benjamini–Hochberg procedure.

To further validate the association between Notch signaling and CB to ICB in relapsed SCLC, we assessed cleaved NOTCH1 expression by IHC across the two ICB-treated cohorts and an additional ICB-treated cohort. The Moffitt cohort consisted of 13 relapsed SCLC patients treated with anti-PD-1 (nivolumab) or anti-PD-L1 (durvalumab) therapy with or without anti-CTLA-4 antibodies (ipilimumab or tremelimumab) (Supplementary Figs. 7 and 8, Supplementary Data 15). Of 59 samples across the cohorts, 32 were evaluable by IHC (NCI cohort, *n* = 6/17; Rochester cohort, *n* = 15/29; Moffitt cohort, *n* = 11/13) (Supplementary Data 1, 9 and 15). Cleaved NOTCH1 was positive (defined as 5% or more positive cells) in a higher percentage of tumors among patients with CB (60% of tumors, *n* = 3/5) than NCB (19% of tumors, *n* = 5/27) (*p* = 0.09) (Supplementary Fig. 9A). Tumors with positive cleaved NOTCH1 staining also had higher expression of the validated Notch signaling targets *REST* (*p* = 0.15) and *NOTCH3* (*p* = 0.17), and higher ssGSEA Hallmark Notch signaling scores (*p* = 0.097) (Supplementary Fig. 9B–D). While these results are not statistically significant, they suggest an overall concordance between Notch signaling assessed by IHC and transcriptome. Altogether, these data demonstrate the heterogeneity of Notch activity in SCLC tumor samples and support the link between Notch activation and immune response.

### Relationship between Notch signaling, neuroendocrine differentiation and immune pathways in SCLC tumors and cell lines

We next sought to determine whether tumors from our ICB-treated SCLC cohorts are comparable to tumors from previously published SCLC datasets with available RNA-seq or microarray data^2,34–37^. We performed principal component analysis of the SCLC cohorts along with cohorts of NE prostate cancer, lung adenocarcinoma and normal prostate using a previously published classifier^38^ (Supplementary Fig. 10). Tumors from both ICB-treated SCLC cohorts from the present study clustered with tumors from two previously published SCLC datasets^2,34^ (Supplementary Fig. 10). In addition, similar to tumors from our ICB-treated cohorts with the highest Notch signaling (based on expression of Hallmark Notch signaling gene set and hereafter referred to as Notch^high^), Notch^high^ tumors and cell lines were not limited to a particular transcriptional subtype (Supplementary Fig. 11 and Supplementary Data 16 and 17).

We next sought to elucidate the characteristics of Notch^high^ tumors and cells. Multivariable analysis across all SCLC tumors revealed a significant negative association between Notch^high^ status and NE score (FDR = 0.003) (Supplementary Fig. 12A–D). Moreover, there was a significant negative relationship between expression of *REST*/*NOTCH3* and NE score (Supplementary Fig. 12E, F). Similarly, Notch^high^ status and *REST*/*NOTCH3* expression were significantly negatively associated with NE score in SCLC cell lines (Supplementary Fig. 13A–F). In addition, Notch^high^ SCLC cells were significantly more likely be categorized as low NE (*p* < 0.001) and to have adherent rather than suspension growth characteristics (*p* < 0.001) (Supplementary Fig. 13G). Thus, NE score was the common, negative predictor of SCLC tumors and cell lines with high Notch signaling.

To further assess the relationship between Notch signaling, NE differentiation and tumor immunity, we segregated tumors from each of the four cohorts into two groups, NE and low NE, by clustering based on NE scores^23^ (Supplementary Fig. 14A) and then assessed for differences in immune pathways. We found significantly higher ssGSEA scores of immune as well as APM and CD8^+^ T-cell signatures in low NE tumors across all four cohorts (Supplementary Fig. 14B). Moreover, multivariable analysis across all SCLC cohorts confirmed the independent, negative association between NE and immune signature scores (Supplementary Fig. 14C).

Given the clear association between NE differentiation and tumor immunity in SCLC tumors, we hypothesized that such a relationship may also exist in extra-pulmonary NE tumors. Recently, a small percentage of TCGA tumors were found to harbor NE properties similar to SCLC, termed “SCN-like”^38^. Using this Pan-Cancer dataset, we found significantly higher immune signature scores in the non-SCN-like compared to the SCN-like tumors (*p* < 0.001) (Supplementary Fig. 14D). There was also a strong negative correlation between NE and immune signature scores across all Pan-Cancer tumors (*R* = −0.49, *p* < 0.001) (Supplementary Fig. 14D). Moreover, the NE score remained a significant negative predictor of immune infiltration (using two independent metrics) after multivariable adjustment for factors previously known to predict tumor immunity such as tumor mutation burden^39^, somatic copy number alterations/aneuploidy^39^ and global methylation^40^ (Supplementary Fig. 14E, F).

Next, we examined whether differences in expression of APM genes based on NE differentiation in SCLC tumors would also apply to SCLC cell lines and mouse models. Of the 66 SCLC cell lines examined^41^, 8 (11%) were classified as low NE (Supplementary Fig. 15A). Similar to the SCLC tumors, low NE cell lines displayed significantly higher expression of APM genes compared to the NE cell lines (*p* = 0.003) (Supplementary Fig. 15B) and the level of NE differentiation correlated with the ssGSEA APM gene set score (*R* = −0.45, *p* < 0.001) (Supplementary Fig. 15C). Both gene set enrichment analysis (GSEA) and gene set variation analysis also revealed enrichment of inflammatory, interferon, immune, and MHC related pathways (FDR = 0) in low NE compared to NE cell lines (Supplementary Fig. 15D and Supplementary Data 17). Within the p53^flox/flox^;Rb^flox/flox^;p130^flox/flox^ conditional triple knockout SCLC mouse model in which green fluorescent protein (GFP) is expressed from the endogenous Hes1 promoter^27^, GFP^high^ cells corresponding to the Notch^high^, low NE phenotype were enriched in inflammatory, immune and antigen presentation pathways compared to GFP^low^ cells consisting of the NE phenotype (FDR = 0) (Supplementary Fig. 15E, F). Thus, these data provide further evidence of the relationship between Notch signaling, NE differentiation, and tumor immunity.

### Overexpression of *NOTCH1* intracellular domain induces a low neuroendocrine phenotype marked by increased expression of APM genes

**Fig. 5:**
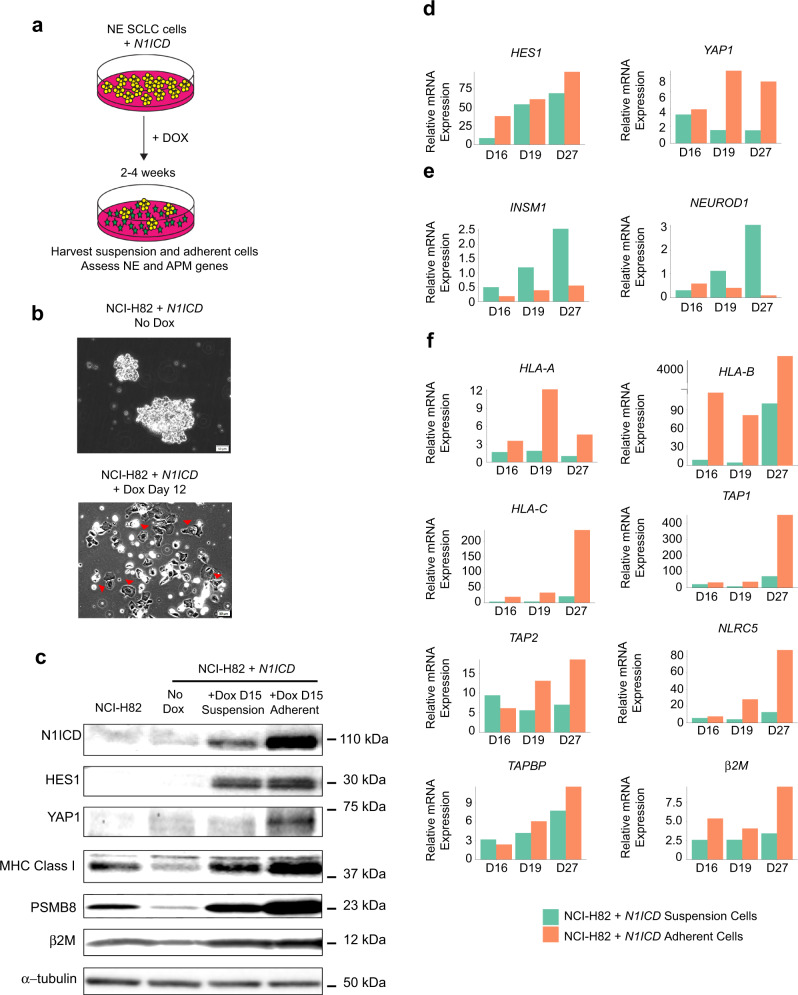
Overexpression of *NOTCH1* intracellular domain in small cell lung cancer induces a high neuroendocrine to low neuroendocrine transition marked by upregulation of antigen processing and presentation genes. **a** Experimental design to assess neuroendocrine (NE) and antigen processing and presentation (APM) markers in suspension and adherent cells after *NOTCH1* intracellular domain (*N1ICD*) overexpression. **b** Brightfield microscopic images of NCI-H82 suspension and adherent cells. Red arrows point to examples of adherent cells. **c** Immunoblot analysis of Notch pathway, NE and immune proteins after *N1ICD* activation for 15 days. Expression of downstream **d** Notch and YAP pathway, **e** NE and **f** APM transcripts. Bars indicate mean of three technical replicates. The data are presented as fold change compared with non-doxycycline treated cells. Three biological replicates at 16, 19, and 27 days after *N1ICD* overexpression are shown. Source data are provided as a Source Data file.

Given our genomic findings, we hypothesized that activation of Notch signaling would downregulate NE differentiation and thereby upregulate APM genes. Thus, to experimentally test the relationship between Notch signaling, NE differentiation and intrinsic tumor immunity, we overexpressed the intracellular, transcriptionally active domain of *NOTCH1* (*N1ICD*) in a NE human SCLC cell line (NCI-H82), which grows in suspension in cell culture. Consistent with previous reports in murine SCLC^2,27^, overexpression of *N1ICD* resulted in the generation of adherent cells starting at ~1 week (Fig. 5a, b). Both suspension and adherent cells were isolated and assessed for expression of NE and APM genes between 2 and 4 weeks after *N1ICD* overexpression (Fig. 5a, b). Suspension cells with high *N1ICD* expression showed higher transcript and protein expression of APM genes compared to control cells, demonstrating that active Notch signaling increases the expression of APM genes (Fig. 5c–f). Strikingly, adherent cells with high *N1ICD* expression, which displayed a low NE phenotype (i.e. low *INSM1*, low *NEUROD1* and high *YAP1* expression), had consistently higher transcript and protein expression of APM genes compared to suspension cells with and without *N1ICD* overexpression (Fig. 5c–f). These experimental data demonstrate that active Notch signaling can upregulate the expression of APM genes in SCLC, particularly when a low NE phenotype is induced.

## Discussion

While immune checkpoint inhibitors are the first class of drugs to be approved for the treatment of SCLC in several decades, the benefits of these drugs are modest in an unselected patient cohort. There is an urgent need to identify SCLC patients who derive meaningful benefit from ICB and to develop rational strategies to augment immunotherapeutic responses. Patients with SCLC, however, rarely undergo tumor biopsies, especially at relapse, thereby limiting studies investigating predictors of response and resistance^42,43^. Our study is unique in that we were able to perform immunogenomic profiling of samples from relapsed SCLC patients treated with ICB. Using two independent ICB-treated cohorts, we discovered and validated an association between high expression of Notch pathway genes and CB to ICB in relapsed SCLC, suggesting Notch signaling as a determinant of CB to ICB treatment. We provide proof-of-concept of Notch activation augmenting critical components of APM that are normally silenced in SCLC^44^, suggesting that modulation of the Notch pathway may be a relevant immunotherapeutic strategy for SCLC.

Notch is an evolutionarily conserved pathway consisting of four receptors (NOTCH1-4) and multiple activating and inhibitory ligands with functional roles in development, transdifferentation, and cell fate that is highly dependent on cellular context and tissue of origin^45^. While Notch has a well-established oncogenic role in cancers such as acute T-cell leukemia^46^, Notch also has a tumour-suppressive role, and is inactivated in the majority of SCLCs^42^. Consistent with previous reports^47^, only a minority of tumors in our ICB-treated SCLC cohorts expressed cleavage-specific NOTCH1. Importantly, however, patients with CB to ICB displayed higher expression of Notch signaling genes. The mechanisms underlying higher Notch activation in select SCLC tumors is unclear. We did not identify differences in *NOTCH* gene mutations or expression of activating or inhibitory Notch ligands in tumors with higher Notch signaling. Increase in Notch signaling upon treatment with drugs that induce histone hyperacetylation or demethylation^48–50^ suggest that epigenetic changes may contribute to the heterogeneity in Notch signaling. Post-translational regulation of Notch receptors and ligands by ubiquitination and E3 ligases such as FBXW7^51^ and Numb^52^ may also alter Notch signaling, but their roles in SCLC are unknown. Further exploration of these and other potential mechanisms regulating Notch signaling in SCLC is warranted.

Although SCLC is clinically treated as a homogenous disease, it has long been known that there are subsets of SCLC tumors such as those with reduced or lack of expression of NE markers^53,54^. Our experimental evidence suggests that Notch activation suppresses NE differentiation and augments tumor-intrinsic immunity in SCLC. These findings are supported by several recent observations. A Pan-Cancer transcriptomic analysis identified a small cell NE phenotype across multiple epithelial tumors characterized by decreased expression of immune and adhesion genes^38^. The loss of MHC-I antigen presentation and components of the APM was also described among EGFR-mutant lung adenocarcinoma with SCLC transformation^55^. In addition, integrative genomic profiling of LCNECs revealed a subset with reduced NE markers and upregulation of immune-related pathways^56^. Despite the relationship between Notch signaling, NE differentiation and tumor immunity, we found only Notch signaling, not NE differentiation, to be significantly associated with CB to ICB. Further investigation into the mechanisms by which Notch signaling may drive tumor immunity, and ultimately response to ICB, will be important for developing future immunotherapeutic strategies in SCLC.

There are several limitations to our study. The discovery cohort was treated with combination ICB (PD-L1 inhibitor plus PARP inhibitor) and therefore the clinical outcomes of patients in this cohort may be confounded by the effects of PARP inhibition. However, findings from our cohort and an independent cohort^57^ suggest no added clinical benefit to combining a PARP inhibitor with ICB compared to historical controls. Our study was also based on cohorts of relapsed SCLC patients whereas ICB is now used in the first-line setting in combination with chemotherapy. Notwithstanding these limitations, our findings provide a basis for future-mechanism-based interventiations to modulate tumor immunogenicity of SCLC patients treated with ICB and chemotherapy in the front-line setting.

In conclusion, while the majority of SCLC patients do not benefit from ICB, in this study, we discover and validate an association between Notch activation and clinical benefit to ICB. Incorporation of gene expression and immunobiological markers may provide the means for more effective application of ICB in patients with SCLC.

## Methods

### Study design

The discovery NCI cohort was a prospective study of relapsed SCLC patients treated on a single-institution, open-label phase II study of anti-PD-L1 antibody durvalumab in combination with olaparib. The primary objective was to determine anti-tumor activity of the combination in patients with relapsed SCLC. Eligible patients were at least 18 years old and had received prior therapy for small cell lung cancer. SCLC was confirmed by pathology review by the Laboratory of Pathology at the National Cancer Institute (NCI) and by a thoracic pathologist from the University of Rochester Medical Center (M.J.V.). CB patients included those with investigator-assessed CR or PR as defined by RECIST 1.1 (i.e., tumor shrinkage >30% from baseline). Patients with brain-only disease progression, but who maintained a systemic response were included in the CB group. NCB patients experienced PD, as determined by RECIST 1.1 and were discontinued from ICB within 8 weeks. All patients provided written informed consent. Patients have consented to sharing indirect identifiers including but not limited to genetic information, race and ethnicity, and sex. The study was approved by the NCI Institutional Review Board with the local protocol number 15C0145. The ClinicalTrials.gov identifier is NCT02484404.

The Rochester validation cohort consisted of relapsed SCLC patients treated with nivolumab. Patients were selected retrospectively based on pharmacy records of nivolumab treatment and a SCLC diagnosis. Patients were further selected based on availability of a tumor biopsy. All patient tumor biopsies were assessed by a thoracic pathologist (M.J.V.) for small cell pathology as well as adequate tissue for molecular analyses. De-identified unstained slides were sent to the NCI. One slide was used for H&E and RNA was extracted from the remaining slides which was used for RNA sequencing and cleaved NOTCH1 staining. CB patients included those with tumor response to nivolumab as determined by the treating oncologist. NCB patients had not evaluable or PD as determined by the treating oncologist. This retrospective study was approved by the University of Rochester Institutional Review Board.

The Moffitt cohort consisted of relapsed SCLC patients previously treated with either ipilimumab and/or nivolumab or tremelimumab and/or durvalumab. Patients were selected retrospectively based on treatment on either study at the Moffitt Cancer Center. Patients were further selected based on protocol requirements and availability of a tumor biopsy. De-identified unstained slides were sent to NCI for patients with available tissue. One slide was used for H&E and additional slides were used for INSM1 and cleaved NOTCH1 staining. This retrospective study was approved by the Moffitt Cancer Center Institutional Review Board.

### Exome and RNA sequencing of tumors

Formalin‐fixed paraffin‐embedded (FFPE) tumor tissue samples were prepared for WES and RNA sequencing (RNA‐Seq). One hundred nanograms of DNA was sheared to approximately 200 base pairs (bp) by sonication (Covaris, Woburn, MA). Exome enrichment was performed using SureSelect Clinical Research Exome Kits according to the manufacturer’s instructions (Aglient, Santa Clara, CA). Paired‐end sequencing (2 × 75 bp) was performed on an Illumina NextSeq500 instrument. The sequences were compared to the human reference genome hg19 using internally developed ClinOmics Somatic Bioinformatic Pipeline v3.1. In brief, raw sequencing data in FASTQ format were aligned against the reference human genome (hg19) with BWA^58^. The Genome Analysis Toolkit and HaplotypeCaller were used for germline single nucleotide variant (SNV) and indel calling; whereas MuTect and Strelka were used for somatic SNV and small indel calling respectively. ANNOVAR was used to functionally annotate genetic variants. Tier 1 somatic variants were defined as protein coding on a hotspot codon or if on a non-hotspot codon then must consist of the following: reported as a somatic change in five or more individual tumors, loss of function in tumor suppressor gene in Cancer Gene Census or loss of function by a known mechanism in a non-tumor suppressor gene for Cancer Gene Census genes. Tier 1 somatic variants were considered “high-confidence” mutations. Other tiers were protein-coding somatic variants not on a hotspot codon but a loss of function variant by an alternative mechanism (Tier 2), a rare/de-novo variant (Tier 3) or not on ClinOmics gene list (Tier 4). FACETS algorithm was used to determine total and allele-specific DNA copy number from WES. Somatic mutational signatures were calculated using deconstructSigs^59^. COSMIC signatures frequently present in lung cancer are shown in Figure1: signature 1 (clock-like), signatures 2 + 3 (APOBEC), signature 3 (defective homologous recombination-based DNA-damage repair), signature 4 (smoking), signature 6 (defective DNA mismatch repair found in microsatellite unstable tumors), signature 15 (associated with defective DNA mismatch repair), signature 24 (cancer samples with known exposures to aflatoxin), signature 29 (found in cancer samples from individuals with a tobacco-chewing habit) and “other” consisting of the remaining signatures.

RNA-seq libraries were prepared using Illumina TruSeq RNA Access Library Prep Kit or TruSeq Stranded mRNA Library Prep Kit according to the manufacturer’s protocol (Illumina) and 75 bp paired-end sequencing was performed using Illumina NextSeq500 sequencers. Sequencer-generated bcl files were converted to fastq files using the bcl2fastq tool in CASAVA (Illumina, San Diego, CA) suite. Paired-end reads were assessed for quality using FastQC. Fastq files were then mapped to GRCh37 reference genome using the STAR/2.5.3a alignment algorithm and subsequently quantified by RSEM program based upon Ensembl GRCh37.75 gene annotation. Read counts for each gene between samples were normalized using TMM method implemented in edgeR and then transformed to RPKM or CPM.

### RNA sequencing data analysis

DESeq2 was used for differential gene expression and the ranked gene list was imported into WEB-based GEne SeT AnaLysis Toolkit (webgestalt.org) which performed GSEA using the Reactome pathway database. ssGSEA from GenePattern (Broad Institute) was also used for Hallmark and Reactome pathways as well as the NE score and immune pathways for each tumor based on previously published gene sets^23,25^. However, the NE gene sets were modified to remove genes that overlapped with other gene sets used for ssGSEA analyses (complete list of genes used for ssGSEA are included in Supplementary Data 18). Heatmaps of transcriptional subtypes were generated using *z*-scores of RNA-seq data (RPKM, FPKM, or CPM) and complete Euclidean linkage parameters. Datasets were combined using *z*-scores of log2 transformed RNA-seq data. Notch^high^ tumors were defined as those above the 75th percentile of ssGSEA Hallmark Notch signaling score within a given dataset. Similarly, for additional analyses, Notch^high^ tumors were defined as those above the 75th percentile expression of *REST* and *NOTCH3*.

### TCR sequencing

The ImmunoSEQ Assay (Adaptive Biotechnologies) covering the CDR3 region of the human TCR β-chain was performed on DNA isolated from baseline and on-treatment blood and select tumor samples. Blood for TCR-seq was collected before treatment, at cycle 1 day 15, and at day 1 of every following cycle. DNA was isolated from peripheral blood mononuclear cells with the Qiagen DNeasy Blood & Tissue Kit. Extracted genomic DNA was amplified in a bias-controlled multiplex PCR, followed by high-throughput sequencing. Sequences were collapsed and filtered to identify and quantitate the absolute abundance of unique TCR-β CDR3 region for further analysis. For alternative TCR-seq of RNA, TCR libraries were prepared using SMARTer Human TCR a/b Profiling Kits according to the manufacturer’s protocol (Takara Bio USA) and 300 bp paired-end sequencing was performed on Illumina MiSeq sequencers. Sequencer-generated bcl files were converted to fastq files using the bcl2fastq tool in CASAVA (Illumina, San Diego, CA) suite. MiXCR 2.1.6 pipeline was used to process raw Fastq data, extract and quantify TCR sequence counts. Measures of population diversity of the T-cell repertoire such as clonality, richness and Jensen-Shannon Divergence index were calculated using a previously published method^60^. Individual T-cell clones were tracked and analyzed using VDJtools^61^.

### NanoString Digital Spatial Profiling

Digital Spatial Profiling (DSP) is based on nCounter^®^ barcoding technology and enables spatially resolved, digital readout of up to 96 proteins or RNA targets in a multiplexed assay. Regions of interest (ROI) of 12 FFPE unstained tissue sections were selected and annotated based on the presence of tumor. The 12 FFPE unstained tissue sections were then stained with 2 fluorescently labeled antibodies and a fluorescent nuclear stain to visualize the tissue morphology. A 31 antibody cocktail panel was applied to the FFPE unstained sections and processed through DSP platform followed by quantitative detection using the nCounter analysis system. For each protein and each sample/patient, the median value across all ROIs was calculated (excluding negative control ROI). The mean of the median was then calculated across all patients. The value of each ROI (excluding negative control ROI) was divided by mean ROI of all the patients. Only ROIs previously annotated as containing sufficient tumor were selected for final analysis.

### External RNA datasets

Four publicly available datasets^2,34,35,37^ were downloaded and data were analyzed in the format received. Raw counts were available for two datasets^2,34^ and trimmed mean of *M*-values (TMM) normalization by EdgeR was used to generate RPKM values. Gene pattern from the Broad Institute was used for single sample GSEA analyses. Gene lists used for analyses are included in Supplementary Data 18. GSEA (ssGSEA) from GenePattern was used to generate Hallmark, Reactome and NE and immune scores for each tumor based on previously published gene sets^23,25^.

### Cell lines and cell culture assays

Human NCI-H82 cells were purchased from the American Type Culture Collection and authenticated by STR testing. All cell lines were grown in RPMI-1640 medium supplemented with 10% fetal bovine serum (Gemini Bio) and 1% penicillin–streptomycin (Gibco). Cells were mycoplasma negative prior to starting the experiments. Cells were virally transduced with a doxycycline-inducible expression of human NOTCH1-ICD plasmid, pLIX-hN1ICD (gift from Julien Sage, Addgene plasmid # 91897). Viral transduction was performed in the presence of polybrene (0.5 ug/ml) and cells were centrifuged at 1200 × *g* for 1.5 h at 30 °C followed by removal of polybrene. After 72 h, cells underwent selection with puromycin (4 μg/ml) for 5 days. Cells were subsequently maintained on puromycin (1 μg/ml). Doxycycline (1 μg/ml) was used induce N1ICD and generate adherent low NE cells from NE cells. After 2–4 weeks of doxycycline exposure, suspension and adherent cells were collected for qRT-PCR analyses and immunoblotting.

### qRT-PCR

RNA was extracted with a Qiagen RNeasy kit and cDNA synthesis was performed with a High Capacity cDNA Reverse Transcription Kit (Applied Biosystems) per the manufacturers’ instructions. Quantitative PCR analysis was performed on a QuantStudio 5 Ssytem with SYBR Green reagents. All samples were assayed in triplicate. Relative expression levels were determined with the ΔΔCt method and normalized to the mean of housekeeping genes GAPDH and B-actin. Primer sequences are available in Supplementary Data 19.

### Immunoblot analysis

Cells were lysed in a modified RIPA buffer (1% NP40, 0.3% SDS, 50 mM Tris-HCl pH 8.0, 150 mM NaCl, 2 mM EDTA, 1% sodiumdeoxycholate, 30 mM NaF, 20 mM Na_4_P_2_O_7_, 1 mM NaVO_3_, 1 mM DTT, 60 mM β -glycerophosphate) supplemented with protease and phosphatase inhibitors. Protein concentration was measured with a Pierce BCA protein assay kit (Thermo Scientific). The antibodies used were cleaved NOTCH1 (CST 4147; dilution 1:1000), HES1 (CST 11988; dilution 1:1000), YAP1 (CST 4912; dilution 1:1000), MHC Class I (Hokudo AB-46-H; dilution 1:10000), PSMB8 (CST 13635; dilution 1:1000), β2M (CST 12851; dilution 1:1000) and α-tubulin (Sigma T9026; dilution 1:15000).

### Immunohistochemical staining and evaluation

Immunohistochemical stains for synaptophysin (790-4407, Roche), chromogranin (760-2519, Roche), CD56 (760-4596, Roche), CD8 (M7103, Dako), INSM1 (sc-271408, Santa Cruz), and Ki-67 (M7240, Dako) were performed at NIH, Laboratory of Pathology, according to the manufacturer’s instruction. Synaptophysin, chromogranin and INSM1 stains were also performed at the University of Rochester Laboratory of Pathology. IHC-stained slides were scanned using the ×40 magnification on NanoZoomer S360 Hamamatsu slide scanner. For INSM1 staining, scoring was performed blinded of clinical data. Nuclear immunoreactivity for INSM1 was considered positive. Histological scores (*H*-scores) were obtained by the formula: 1 × (% of 1+ cells) + 2 × (% of 2+ cells) + 3 × (% of 3+ cells).

For cleaved NOTCH1 IHC staining, 4–5 μm tissue sections were baked for 1 h at 60 °C, deparaffinized in xylene, and rehydrated through a series of graded alcohol to distilled water. Heat-mediated antigen retrieval was then performed in a pressure chamber (Pascal; Dako, Carpinteria, CA) with pH 9 EDTA buffer (Dako). Endogenous peroxidase activity was quenched using a 3% solution of aqueous hydrogen peroxide. Subsequently, primary antibody hybridization was carried out with the Rabbit monoclonal anti-cleaved NOTCH1 (Val1744-D3B8; diluted 1:50; Cell Signaling Technology #4147) for 1 h at room temperature. Signals were detected with an Envision+Rb detection system (Dako) and visualized using 3,3′-diaminobenzidine, lightly counterstained with hematoxylin, dehydrated in ethanol, cleared in xylene, and coverslipped. Normal skin samples were included as positive controls. Anti-cleaved NOTCH1 IHC was scored blinded to clinical data.

### Quantification and statistical analysis

All figures and graphs were generated using the “ggplot2” package available through the R statistical program. Correlations, t-tests, and regressions were conducted though the R base packages. All correlations shown are Pearson correlation coefficients. All tests were two-tailed and p-values less than 0.05 were considered significant.

### Reporting summary

Further information on research design is available in the Nature Research Reporting Summary linked to this article.

## Supplementary information

Supplementary Information

Description of Additional Supplementary Files

Dataset 1-7

Dataset 8

Dataset 9-13

Dataset 14

Dataset 15

Dataset 16

Dataset 17

Dataset 18

Dataset 19

Reporting Summary

## Data Availability

Raw exome, RNA sequencing and RNA-based TCR sequencing data generated in this study have been deposited in dbGaP under accession code phs002176. Raw DNA-based TCR sequencing data from Adaptive Biotech is not available due to company restrictions. All unanalyzed DNA and RNA-based TCR sequencing data can be retrieved from: https://github.com/nitinroper/SCLC-ICB-NCI. Previously published small cell lung cancer RNA sequencing datasets used in this manuscript are available in dbGaP under accession code phs001049.v1.p1, Gene Expression Omnibus under accession codes GSE60052 and GSE43346, European Genome-Phenome Archive under accession code EGAS00001000925. Source data are available as a Source Data file. The remaining data are available within the article, Supplementary Data and Supplementary Information. Source data are provided with this paper.

## Source data

Source Data
