# Supplementary material for: Notch signaling and efficacy of PD-1/PD-L1 blockade in relapsed small cell lung cancer
Source: Nat Commun. 2021 Jun 23;12:3880. doi: 10.1038/s41467-021-24164-y (PMC8222224; doi:10.1038/s41467-021-24164-y)
Supplement: Supplementary file 1 — Supplementary Information [file 41467_2021_24164_MOESM1_ESM.pdf]

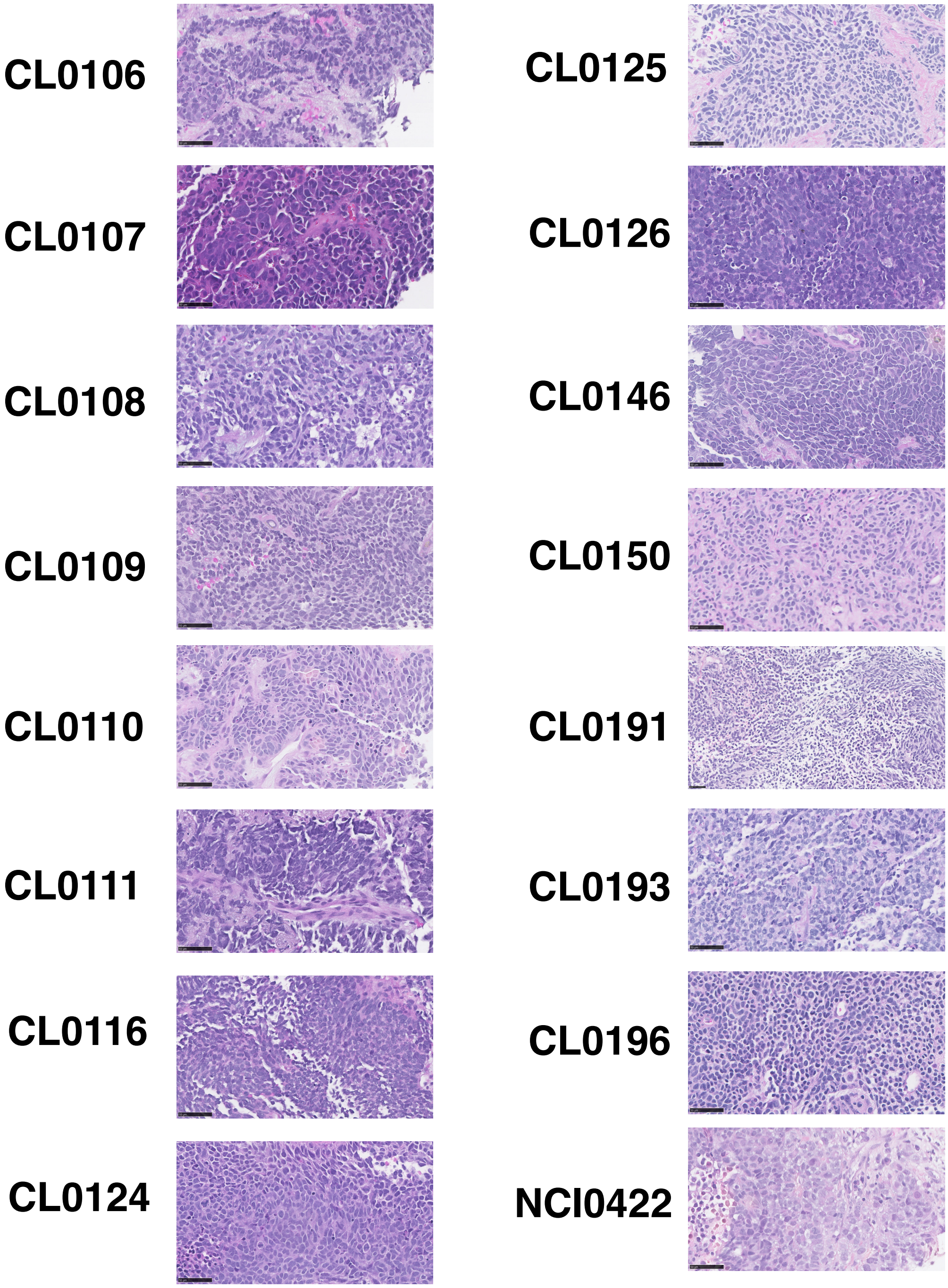

Figure S1: H&E images of tumors in NCI discovery cohort of patients treated with combination ICB. Tumor IDs are listed to the left of each H&E image. Scale bars, 50 µm. H&Es for CL0114 and CL0147 are not shown.

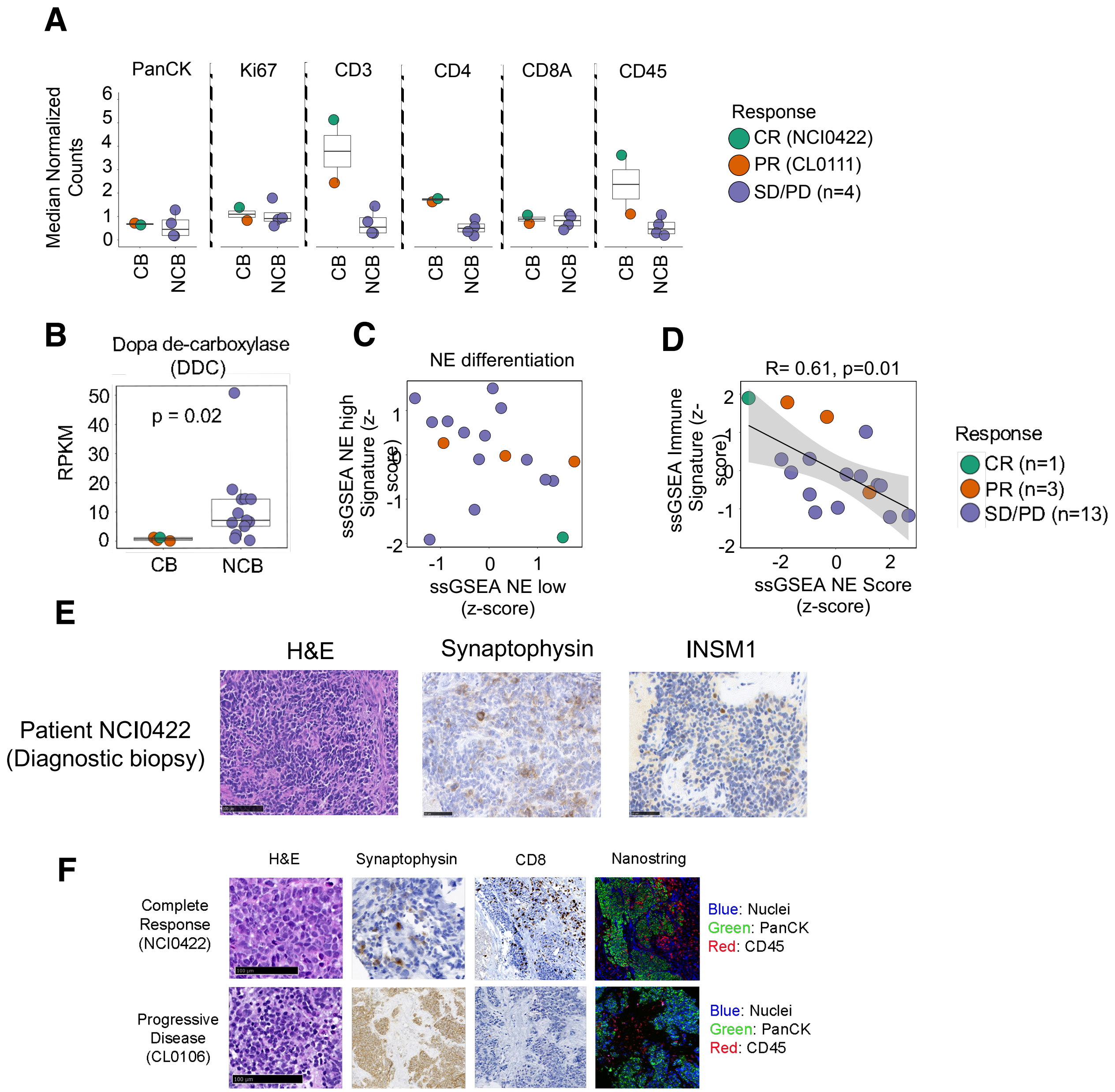

Figure S2: Transcriptomic and immunohistochemical markers of tumor immunity and neuroendocrine differentiation among NCI discovery cohort patients treated with combination ICB. (A) Digital spatial profiling of B and T-cell antigens in tumors of patients with and without CB to ICB. **(B)** Expression of NE gene dopa de-carboxylase (*DDC*) in tumors of patients with CB and NCB. **(C)** ssGSEA NE differentiation scores of all tumors. **(D)** Correlation between ssGSEA NE differentiation and immune gene set scores across all tumors. 95% confidence interval for the line shown in grey shading. **(E)** IHC staining for synaptophysin and INSM1 are shown for a diagnostic biopsy in patient NCI0422. Scale bars, 100 μm for H&E and 50 μm for synaptophysin and INSM1. **(F)** H&E, Synaptophysin and CD8 IHC for a patient with complete response (NCI0422) and a patient with progressive disease (CL0108). Scale bars, 100 μm Digital spatial profiling (Nanostring) image is also shown. Statistical significance was tested with a two-tailed Mann–Whitney U-test. Blue: Nuclei; Green: PanCK; Red: CD45. Boxplots represent the median, 25th and 75th percentiles and the vertical bars span the 5th to the 95th percentiles. Source data are provided as a Source Data file

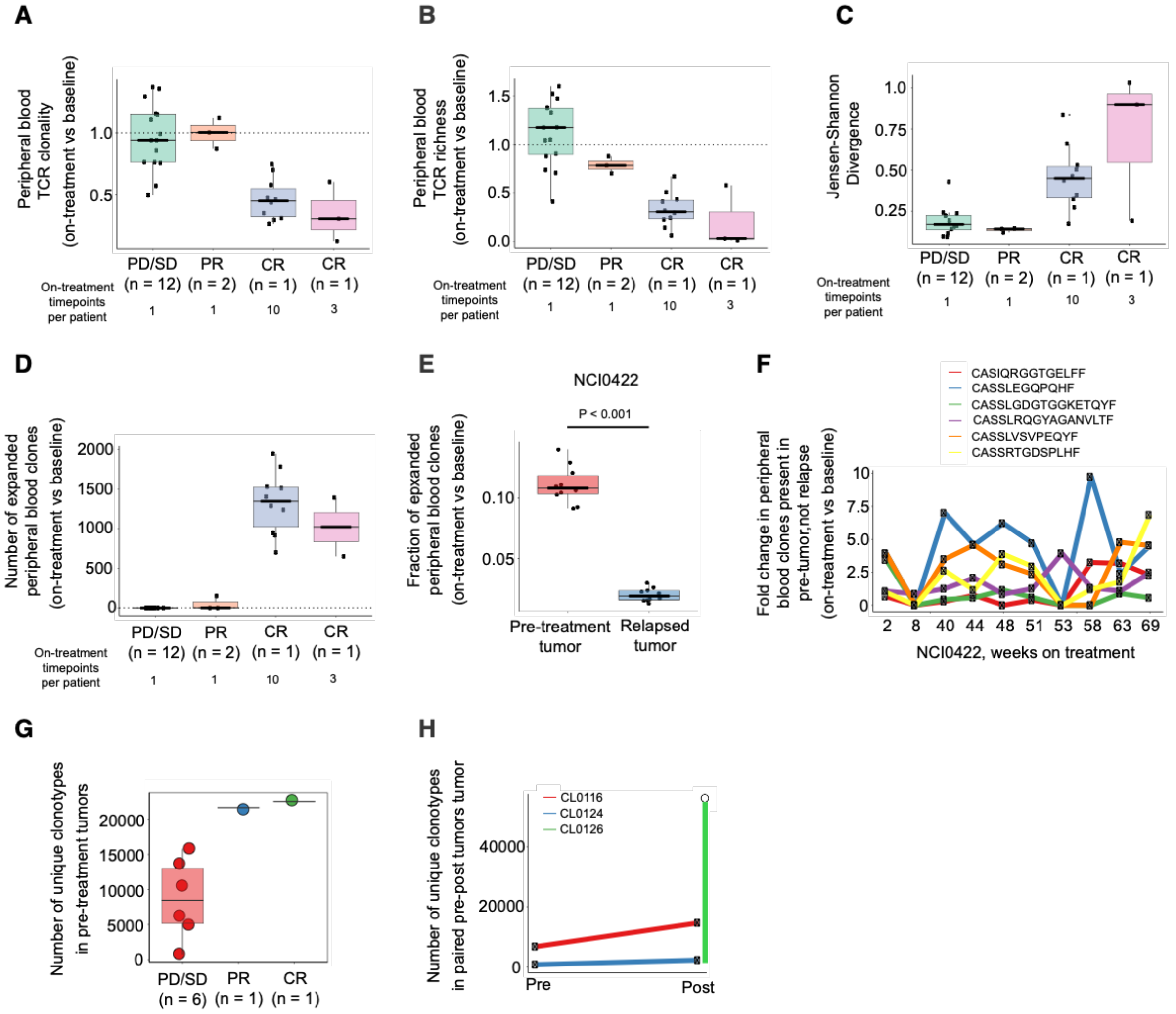

Figure S3: Alterations in peripheral blood T-cell repertoire and T-cell clonotype expansion are associated with clinical benefit to combination ICB in NCI discovery cohort. Fold change in peripheral blood T-cell receptor **(A)** clonality, **(B)** richness, and **(C)** Jensen-Shannon Divergence in on-treatment with combination ICB versus pre-treatment samples. Patients are stratified by response. Additional on-treatment timepoints (blue box plots) and an alternative RNA-based TCR-seq (pink box plots) were analyzed for patient NCI0422 (CR). Dotted black line indicates no change. **(D)** Number of expanded peripheral blood clones between on-treatment and pre-treatment samples. Expanded clones are defined as unique, productive clonotypes with fold change greater than 3 and greater than 10 total reads from TCR-sequencing of peripheral blood. **(E)** Fraction of expanded peripheral blood clones from each timepoint of patient NCI0422 present in pre-treatment tumor and tumor at relapse. **(F)** Fold change in expanded peripheral blood clones of patient NCI0422 across multiple timepoints present in the pre-treatment tumor but not in the tumor at relapse. CDR3 amino acid sequences for each of the six peripheral blood clones are shown. **(G)** Number of unique, productive clonotypes within pre-treatment tumor biopsies. Boxplots represent the median, 25th and 75th percentiles and the vertical bars span the 5th to the 95th percentiles. **(H)** Number of unique, productive clonotypes in paired pre-post tumors. Post only tumor is shown for patient CL0126 (PR). Statistical significance was tested with a two-tailed Mann–Whitney U-test (unadjusted p-values shown). PD: progressive disease; SD: stable disease; PR: partial response; CR: complete response. Source data are provided as a Source Data file.

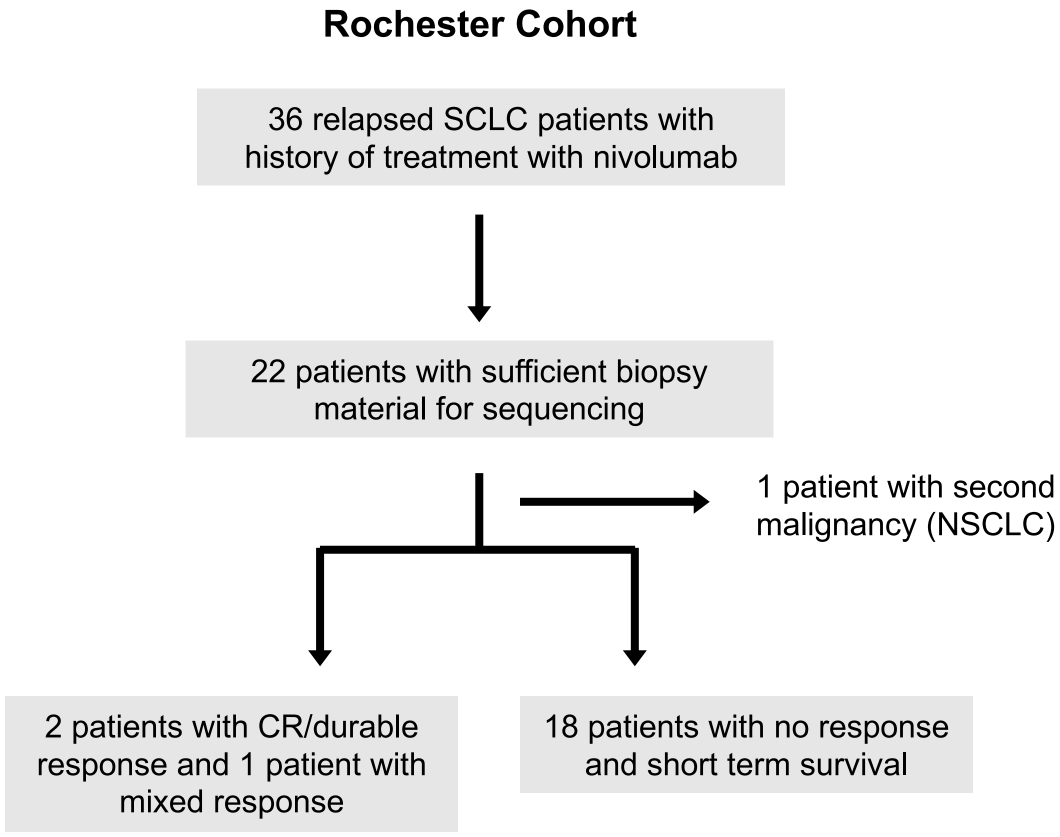

Figure S4: Flow diagram of samples from the Rochester validation cohort of relapsed SCLC patients treated with ICB.

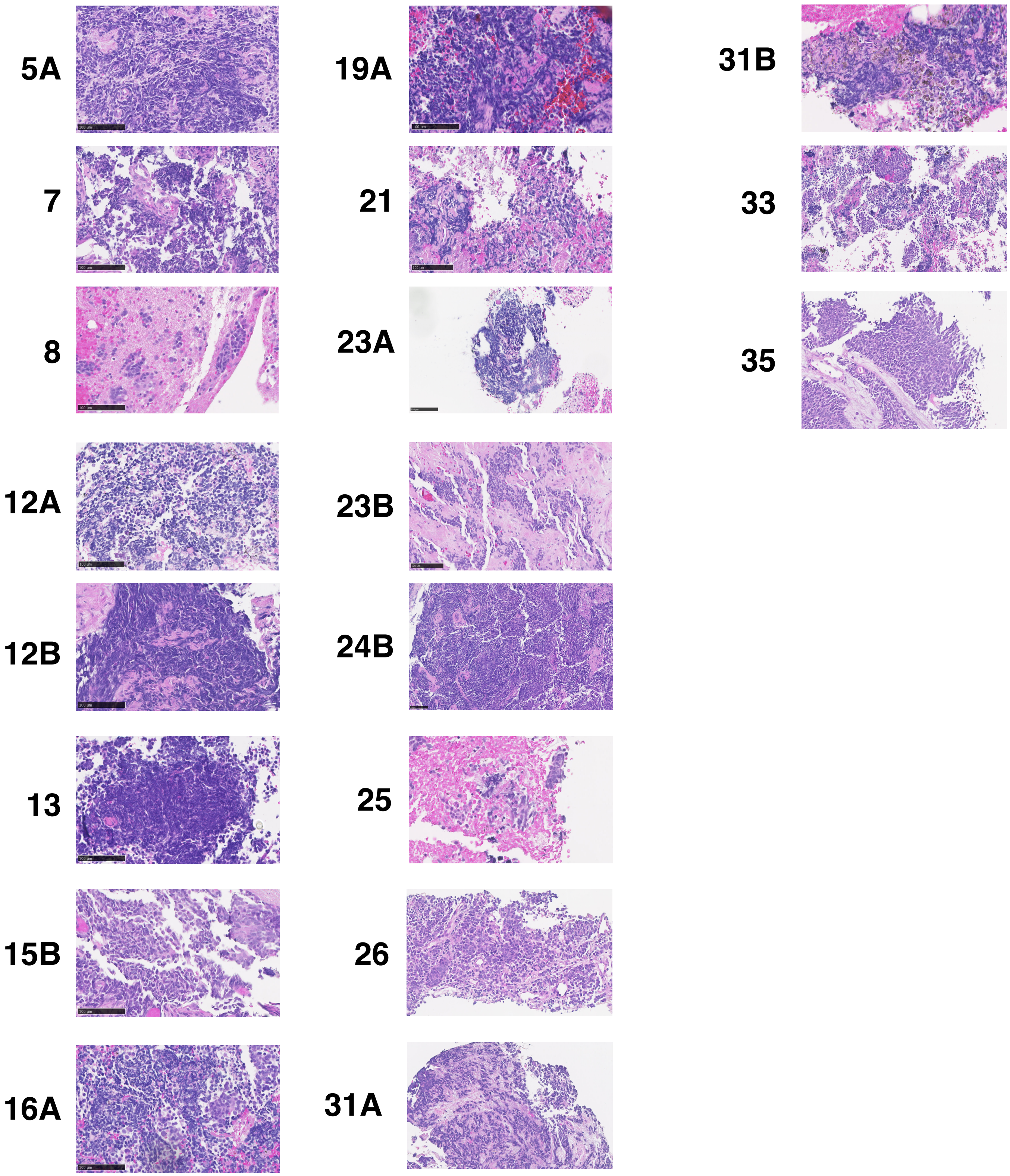

Figure S5: H&E images of tumors in Rochester validation cohort of relapsed SCLC patients treated with ICB. Tumor IDs are listed to the left of each H&E image. Scale bars, 100 µm. H&Es for 2, 9, 14, 17 and 18 are not shown.

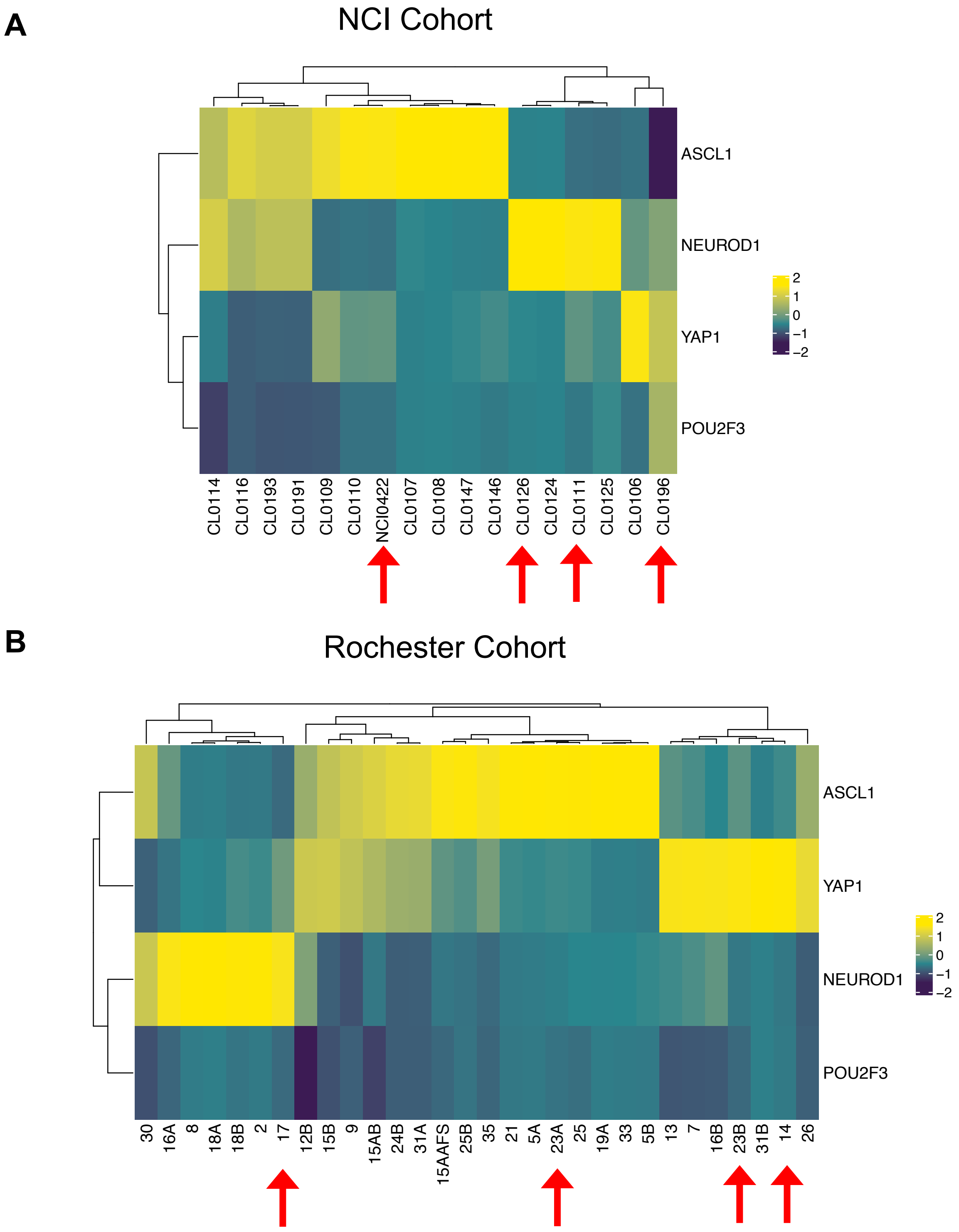

Figure S6: Transcriptional subtypes based on lineage oncogenes of ICB treated SCLC cohorts. Hierarchical clustering of *ASCL1*, *NEUROD1*, *POU2F3* and *YAP1* gene expression in two ICB treated SCLC cohorts: **(A)** NCI discovery cohort and **(B)** Rochester validation cohort**.** Relative gene expression is shown (z-score). Red arrows point to tumors from patients who derived clinical benefit to ICB. Source data are provided as a Source Data file.

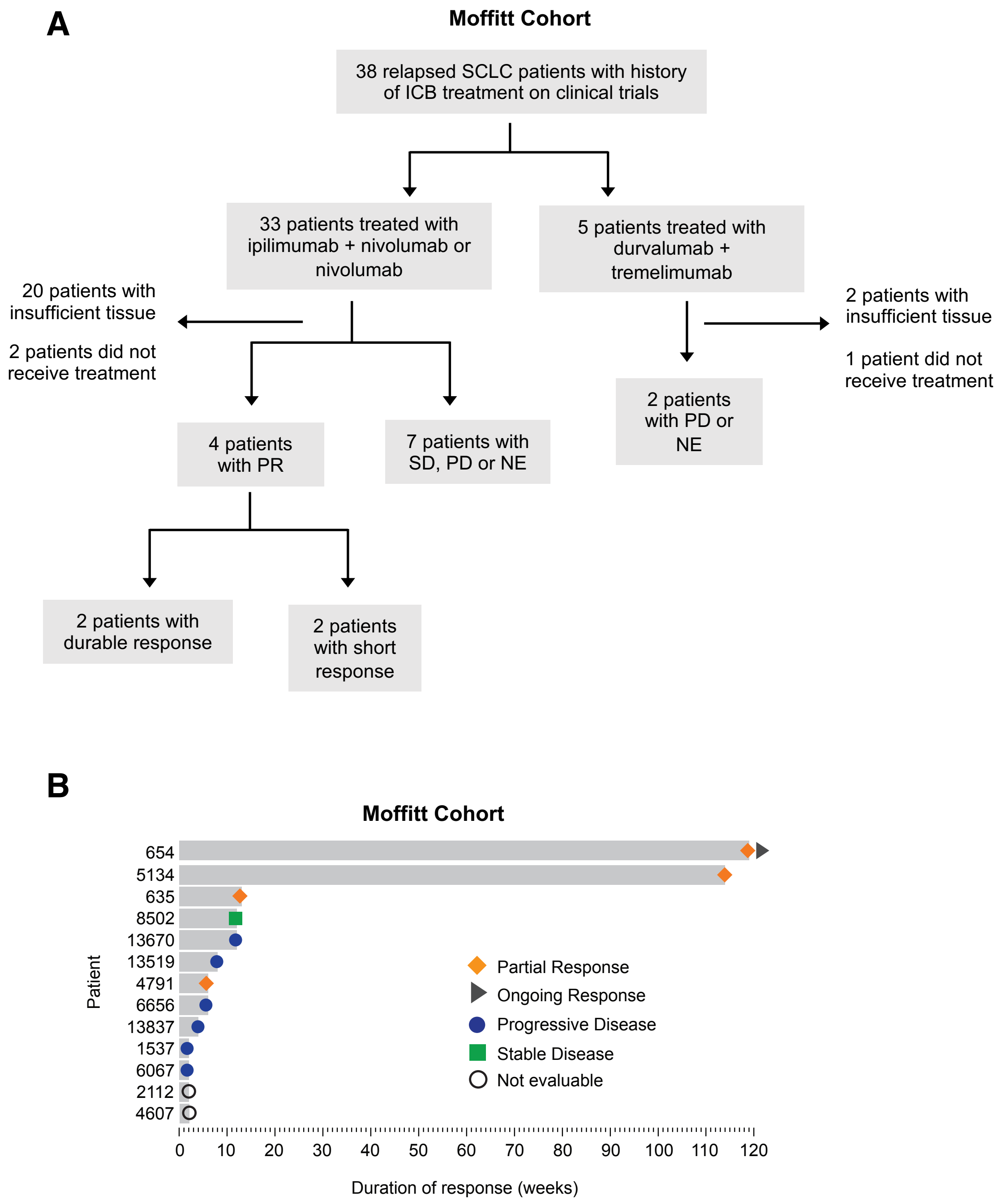

Figure S7: Flow diagram of samples and clinical responses of patients in Moffitt cohort of relapsed SCLC patients treated with ICB. (A) Flow diagram of samples in Moffitt Cohort. (**B**) Swimmer's plot indicating response and length of time on therapy for individual patients in the Moffitt cohort. SD: stable disease; PD: progressive disease; PR: partial response; NE: Not evaluable disease

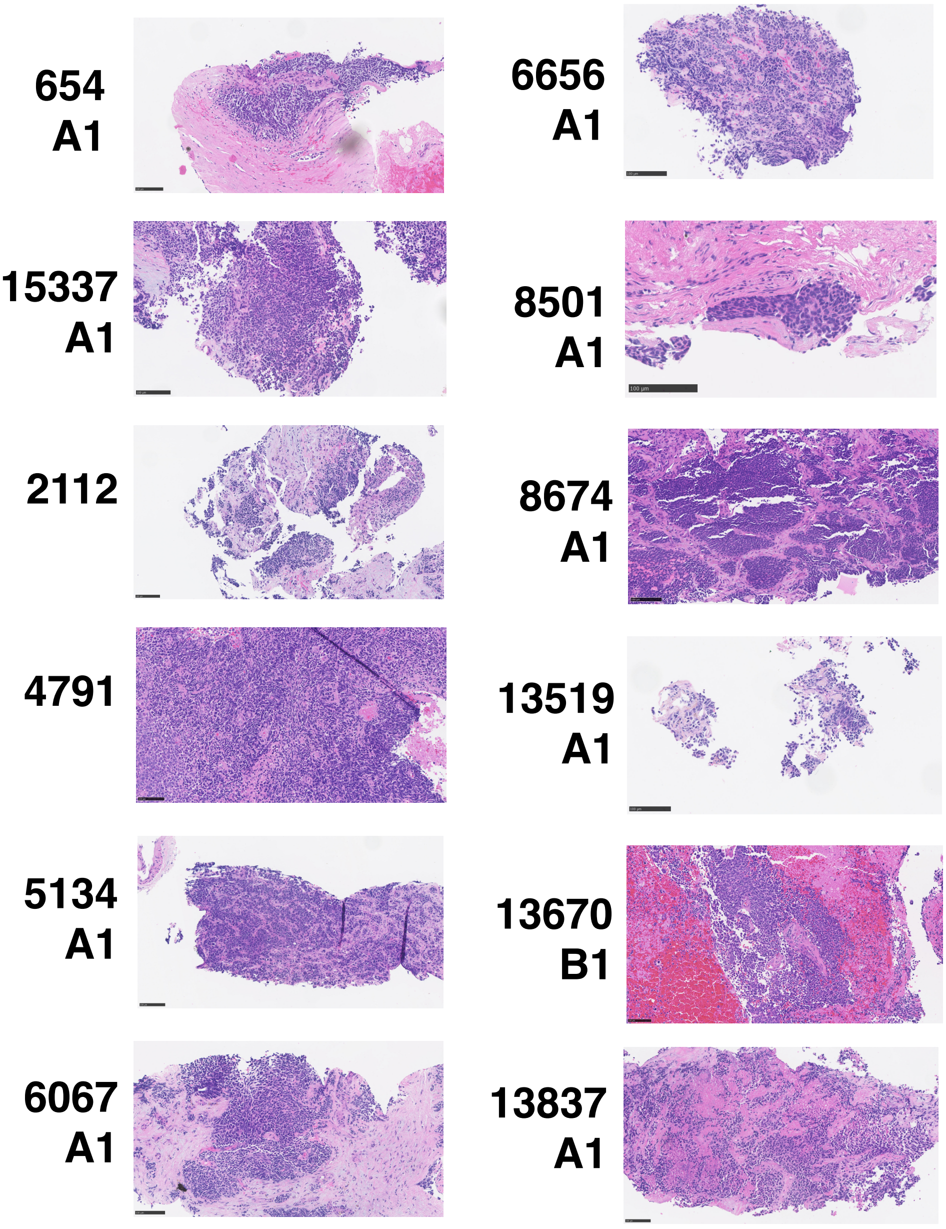

Figure S8: H&E images of tumors in Moffitt cohort of relapsed SCLC patients treated with ICB. Tumor IDs are listed to the left of each H&E image. Scale bars, 100 μm. H&E for 4607 is not shown.

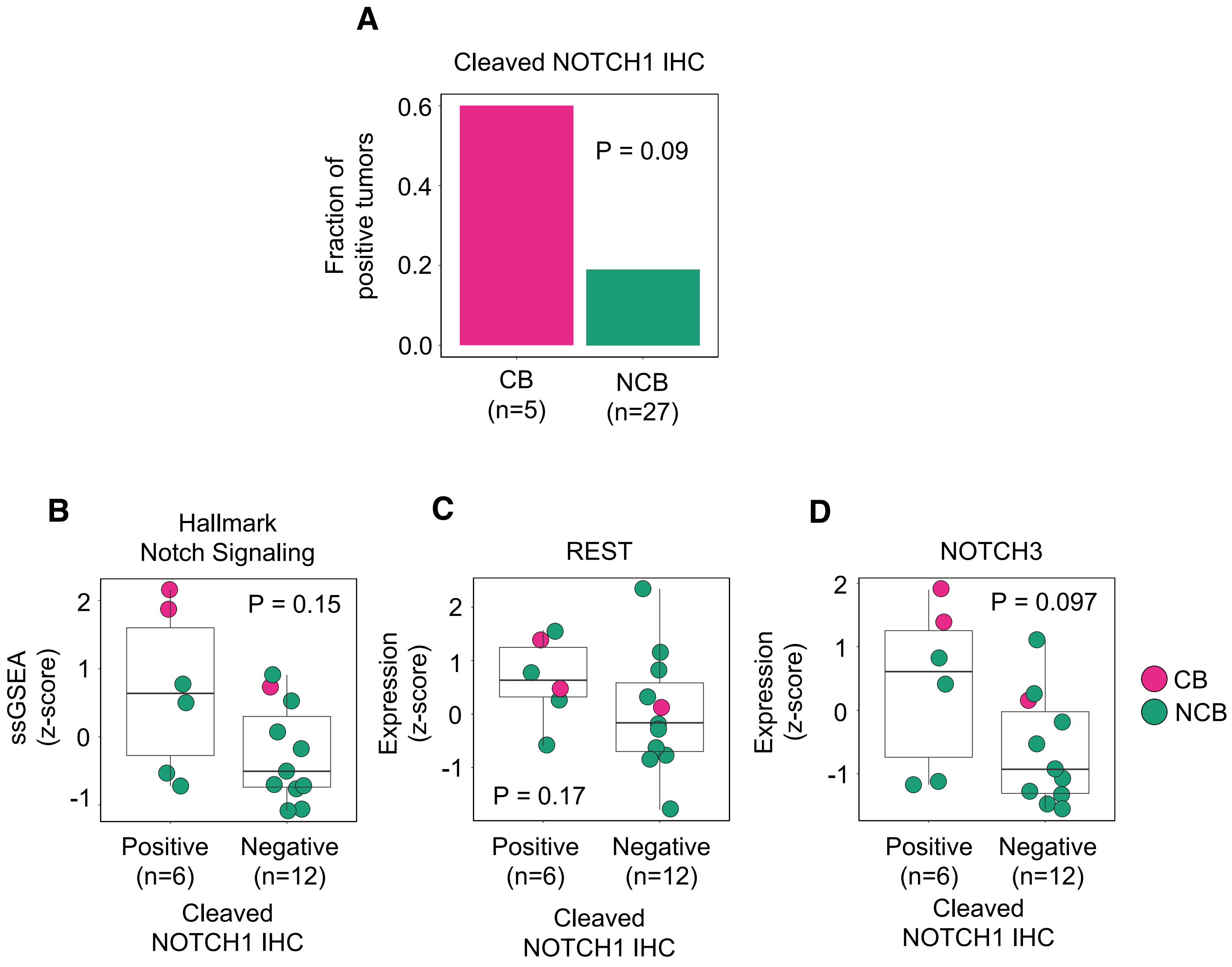

Figure S9: Cleaved NOTCH1 immunohistochemistry across ICB treated cohorts of relapsed SCLC. (A) Fraction of tumors with positive cleaved NOTCH1 IHC (defined as 5% or more positive cells) stratified by CB or NCB to ICB across three ICB-treated cohorts (NCI, Rochester and Moffitt). P-values calculated by Fisher's two-tailed exact test. **(B)** ssGSEA score for Hallmark Notch signaling, **(C)** *REST* expression and **(D)** *NOTCH3* expression of tumors in NCI and Rochester cohorts stratified by positive or negative cleaved NOTCH1 IHC. Statistical significance was tested with a two-tailed Mann–Whitney U-test. Boxplots represent the median, 25th and 75th percentiles and the vertical bars span the 5th to the 95th percentiles. Colored circles represent either CB (pink) or NCB (green) to ICB. CB: clinical benefit; NCB: no clinical benefit. IHC: immunohistochemistry. Source data are provided as a Source Data file.

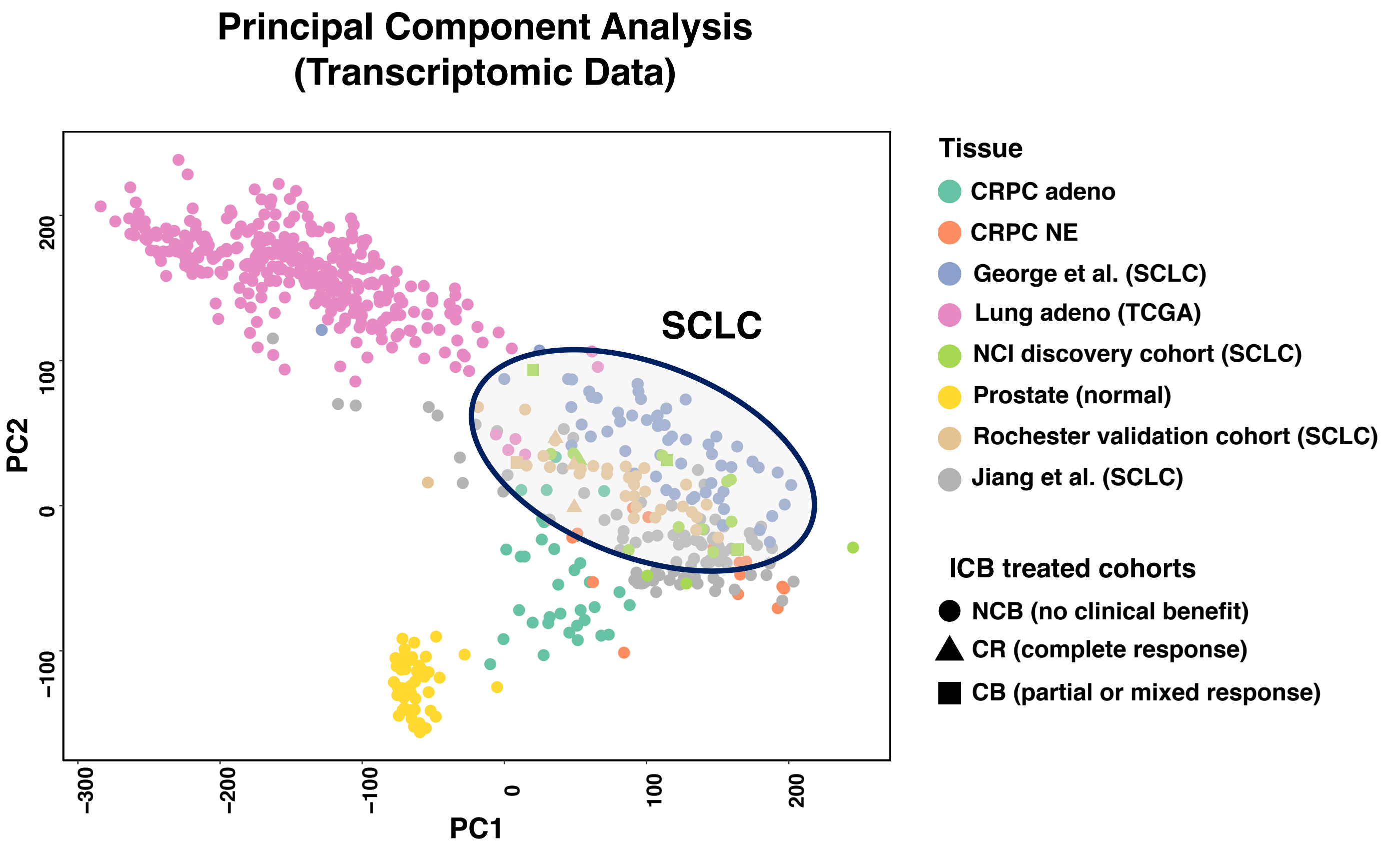

Figure S10: Transcriptomic relationship between ICB treated SCLC cohorts with previously published non-ICB treated SCLC, lung adenocarcinoma, and prostate cancer cohorts. Ellipse demarcates SCLC tumors from all cohorts. Source data are provided as a Source Data file.

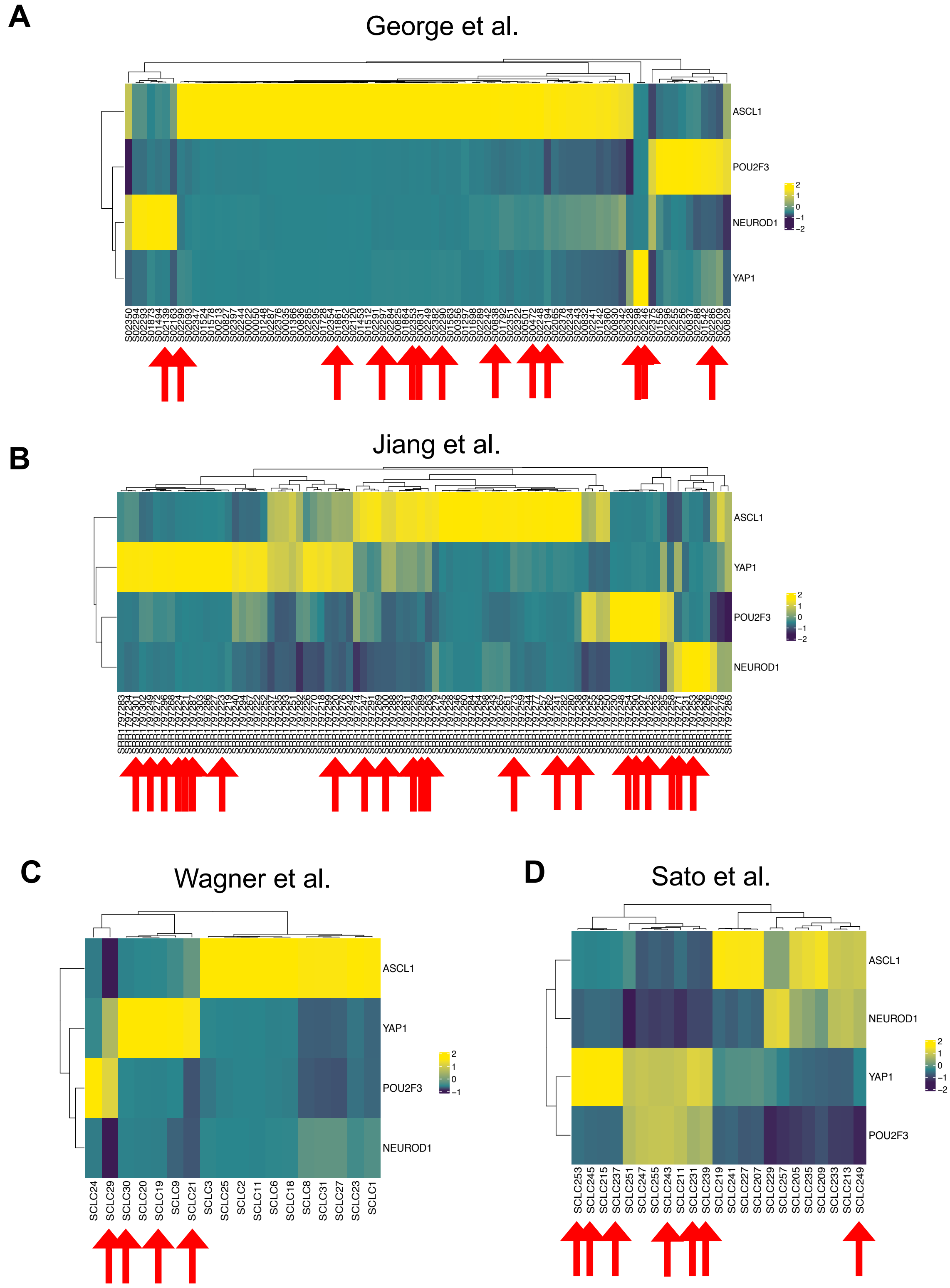

Figure S11: Transcriptional subtypes based on lineage oncogenes of non-ICB treated SCLC cohorts. Hierarchical clustering of *ASCL1*, *NEUROD1*, *POU2F3* and *YAP1* gene expression in four small cell lung cancer cohorts without ICB treatment: **(A)** George et al., **(B)** Jiang et al., **(C)** Wagner et. al. and **(D)** Sato et al. Relative gene expression is shown (z-score). Red arrows point to tumors that have high Hallmark Notch gene set expression (Notch^{high}). Source data are provided as a Source Data file.

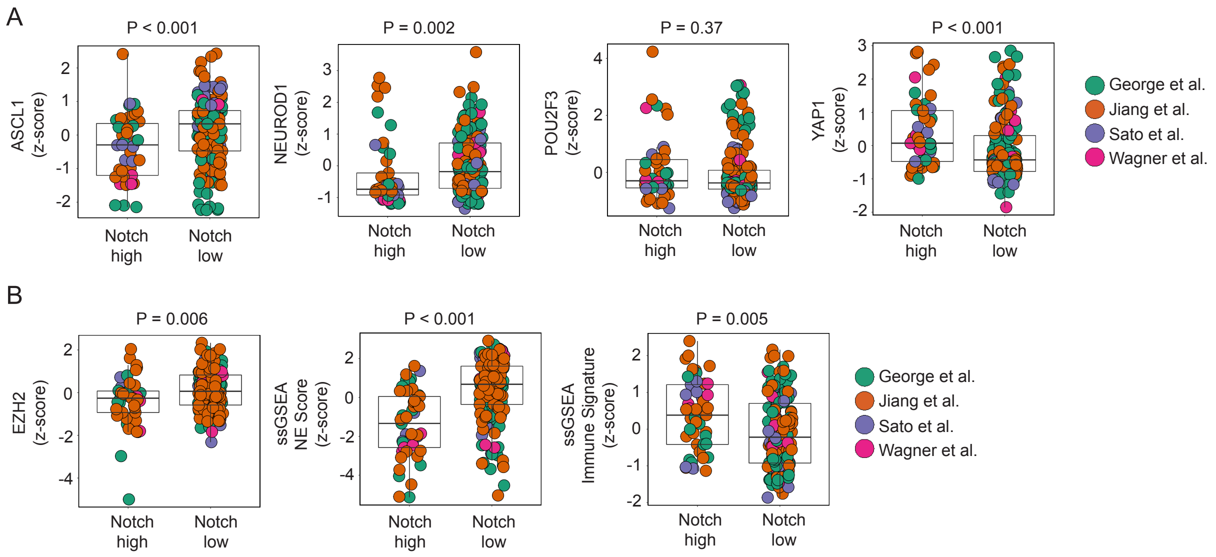

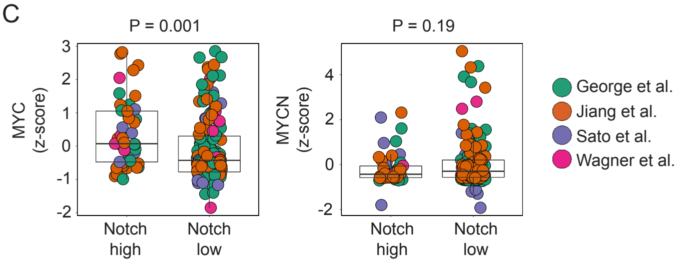

D

Multivariable analysis

Variable	Estimate	t-value	p-value	FDR
NE Score	-0.09	-3.54	0.0005	0.003
EZH2 expression	-0.05	-1.50	0.13	0.30
Immune Signature	-0.05	-1.35	0.18	0.30
MYC expression	0.01	0.42	0.67	0.83
NEUROD1 expression	0.0009	0.03	0.98	0.98

Outcome (dependent) variable = ssGSEA Hallmark Notch Signaling

E

Multivariable analysis

Variable	Estimate	t-value	p-value	FDR
NE Score	-0.11	-4.274	0.00003	0.0002
EZH2 expression	0.0098	0.303	0.76	0.015
Immune Signature	-0.11	-2.754	0.006	0.47
MYC expression	0.036	1.094	0.28	0.85
NEUROD1 expression	0.0057	0.184	0.85	0.85

Outcome (dependent) variable = NOTCH3 expression

F

Multivariable analysis

Variable	Estimate	t-value	p-value	FDR
NE Score	-0.14	-6.512	$5.75e^{-10}$	$5.75e^{-09}$
EZH2 expression	0.073	2.769	0.0062	0.016
Immune Signature	0.0037	0.107	0.92	0.27
MYC expression	0.0084	0.309	0.76	0.92
NEUROD1 expression	-0.037	-1.419	0.16	0.92

Outcome (dependent) variable = REST expression

Figure S12: Notch signaling is significantly associated with neuroendocrine differentiation across non-ICB treated SCLC cohorts. Association between expression of Hallmark Notch signaling gene set stratified by high (n=45) and low (n=163) and (**A**) expression of lineage oncogenes *ASCL1*, *NEUROD1*, *POU2F3* and *YAP1;* (**B**) NE score, expression of immune gene set and expression of *EZH2;* and **(C)** expression of *MYC* and *MYCN*. (**D**) Multivariable analysis of variables associated with ssGSEA Hallmark Notch gene set score and with expression of validated Notch signaling target genes **(E)** *NOTCH3* and **(F)** *REST*. P-values shown were calculated using a two-tailed Wilcoxon signed-rank test. Linear regression was used for multivariable analysis. False discovery rate was calculated using the Benjamini-Hochberg procedure. Boxplots represent the median, 25th and 75th percentiles and the vertical bars span the 5th to the 95th percentiles. Source data are provided as a Source Data file.

A

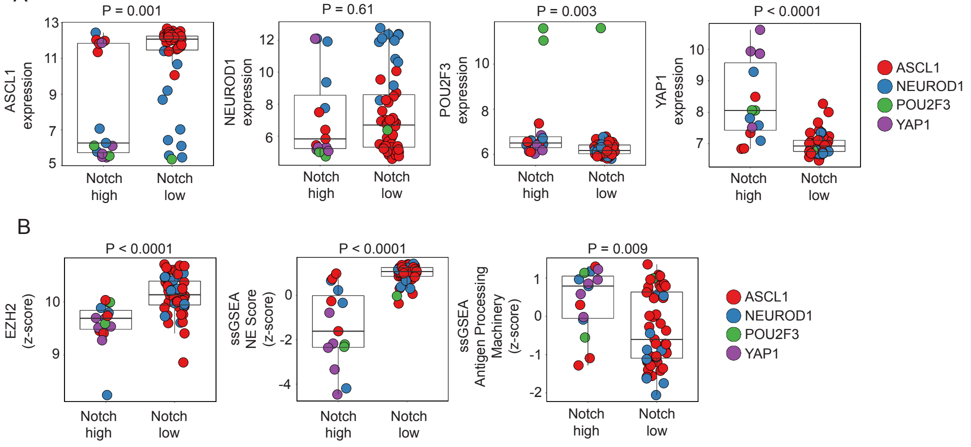

C

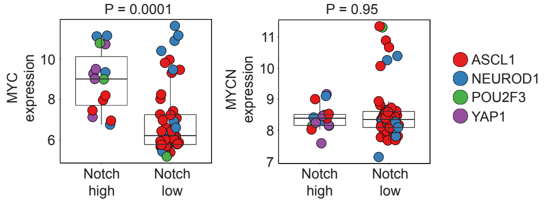

D

Multivariable analysis

Variable	Estimate	t-value	p-value	FDR
NE Score	-0.22	-5.94	$1.53x10^{-7}$	$6.5x10^{-7}$
EZH2 expression	-0.23	-2.63	0.011	0.02
POU2F3 expression	0.019	0.59	0.56	0.47
MYC expression	0.006	0.28	0.78	0.47
APM signature	-0.017	-0.43	0.67	0.54

Outcome (dependent) variable = ssGSEA hallmark Notch signaling

E

Multivariable analysis

Variable	Estimate	t-value	p-value	FDR
NE Score	-0.20	-6.45	$2.15x10^{-8}$	$1.8x10^{-7}$
EZH2 expression	0.13	1.69	0.096	0.125
POU2F3 expression	0.093	3.40	0.0012	0.003
MYC expression	0.031	1.648	0.10	0.125
APM signature	0.014	0.43	0.668	0.668

Outcome (dependent) variable = REST expression

F

Multivariable analysis

Variable	Estimate	t-value	p-value	FDR
NE Score	-0.139	-3.32	0.0016	0.008
EZH2 expression	-0.018	-0.175	0.86	0.88
POU2F3 expression	-0.005	-0.146	0.88	0.88
MYC expression	-0.023	-0.905	0.37	0.62
APM signature	0.043	0.966	0.34	0.62

Outcome (dependent) variable = NOTCH3 expression

G

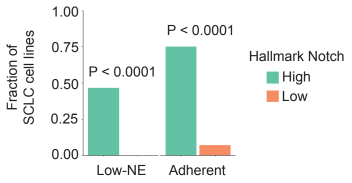

Figure S13: Notch signaling is significantly associated with neuroendocrine differentiation across SCLC cell lines. Association between expression of Hallmark Notch signaling gene set stratified by high (n=45) and low (n=163) and (**A**) expression of lineage oncogenes *ASCL1*, *NEUROD1*, *POU2F3* and *YAP1;* (**B**) expression of EZH2, NE score score, and Antigen Processing Machinery score*;* and **(C)** expression of *MYC* and *MYCN*. (**D**) Multivariable analysis of variables associated with ssGSEA Hallmark Notch gene set score and with expression of validated Notch signaling target genes **(E)** *NOTCH3* and **(F)** *REST*. (**G**) Association between expression of Hallmark Notch signaling gene set (stratified by high and low) with low-NE classification and adherent cell culture properties. P-values shown were calculated using a two-tailed Wilcoxon signed-rank test. Linear regression was used for multivariable analysis. False discovery rate was calculated using the Benjamini-Hochberg procedure. Boxplots represent the median, 25th and 75th percentiles and the vertical bars span the 5th to the 95th percentiles. Source data are provided as a Source Data file.

A B

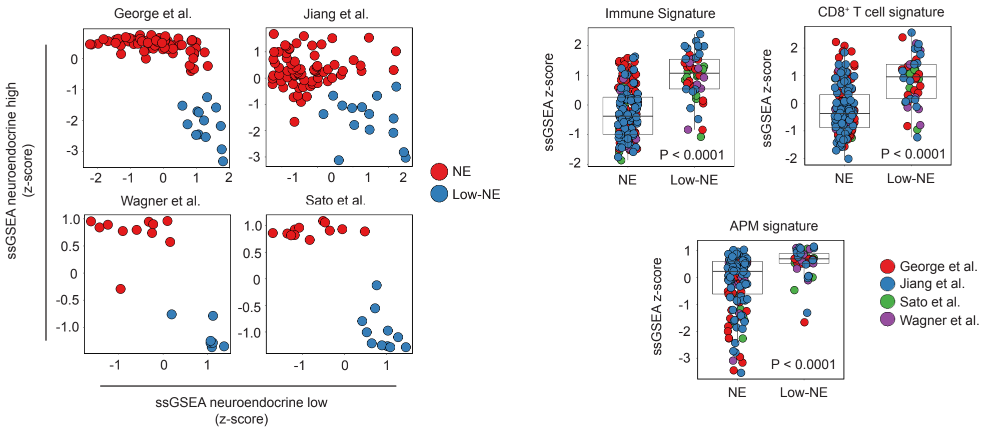

C

Multivariable analysis
(SCLC cohorts)

Variable	Estimate	t-value	p-value	FDR
NE Score	-0.39	-11.58	$<2x10^{-16}$	$6x10^{-16}$
EZH2 expression	-0.10	-1.96	0.05	0.18
MYC expression	-0.05	-0.85	0.37	0.40

Outcome (dependent) variable = ssGSEA Immune Signature

D

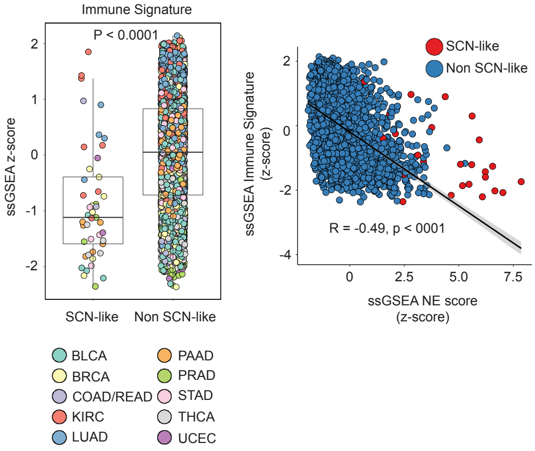

E

Multivariable analysis
(Pan Cancer)

Variable	Estimate	t-value	p-value
NE Score	-0.33	-38.61	$<2x10^{-16}$
Total mutations[2]	$8.22x10^{-5}$	3.53	$4.2x10^{-4}$
Somatic copy number[2]	-0.05	-6.12	$1.2x10^{-9}$
Global methylation[3]	4.8	20.51	$<2x10^{-16}$

Outcome (dependent) variable = ssGSEA Immune Signature

F

Multivariable analysis
(Pan Cancer)

Variable	Estimate	t-value	p-value
NE Score	-0.02	-7.69	$<2x10^{-14}$
Total mutations[2]	$2.1x10^{-5}$	2.41	0.02
Somatic copy number[2]	-0.02	-6.19	$7.6x10^{-10}$
Global methylation[3]	0.9	11.08	$<2x10^{-16}$

Outcome (dependent) variable = Immune Signature Score[2]

[2]Davoli et al.

[3]Jung et al.

Figure S14: Neuroendocrine differentiation is significantly associated with expression of immune gene sets in published SCLC cohorts and Pan-Cancer. (**A**) Segregation of tumors into NE (n=166) and low-NE (n=42) phenotypes in each of four SCLC cohorts. (**B**) ssGSEA antigen processing machinery, CD8+ T-cell and immune signature scores in NE and low-NE tumors across the four SCLC cohorts. (**C**) Multivariable analysis of variables associated with ssGSEA immune signature score across four SCLC cohorts. (**D**) ssGSEA immune signature score between Pan-Cancer SCN-like (n=45) and non-SCN-like (n=4660) tumors and correlation between ssGSEA NE and ssGSEA immune signature scores. (**E and F**) Multivariable analysis of factors associated with expression of ssGSEA immune signature score Pan-Cancer. P-values shown were calculated by Wilcoxon signed-rank test. Linear regression was used for multivariable analysis. False discovery rate was calculated using the Benjamini-Hochberg procedure. Boxplots represent the median, 25th and 75th percentiles and the vertical bars span the 5th to the 95th percentiles. Source data are provided as a Source Data file.

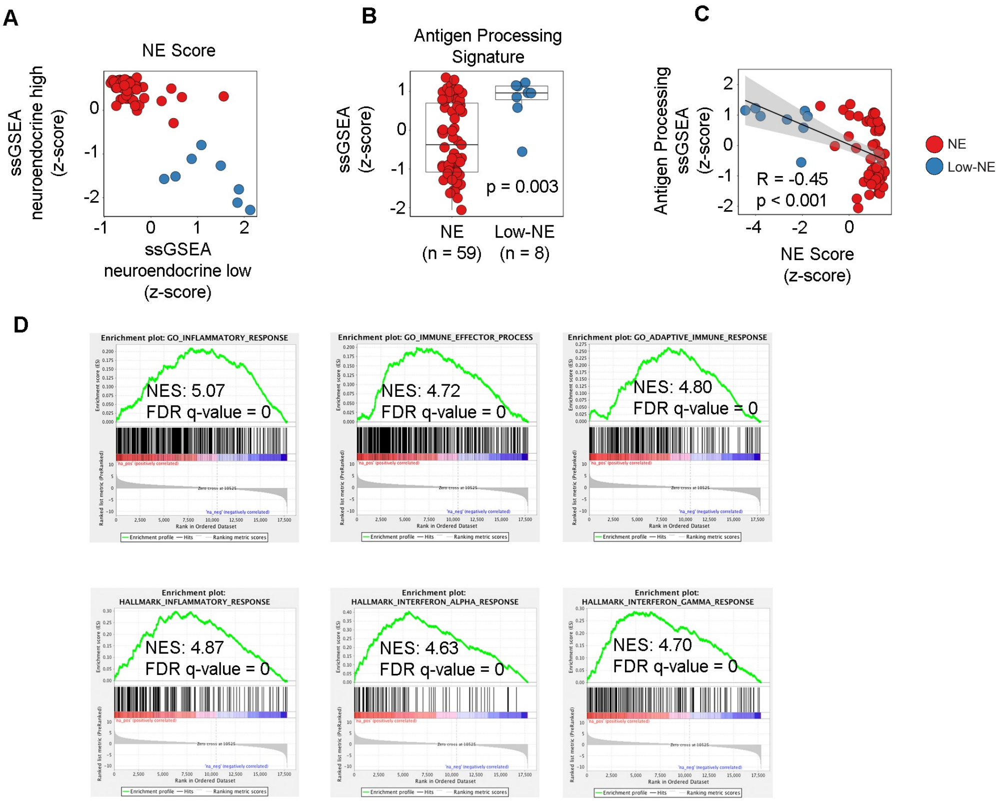

E

GSEA for Hallmark signature genes sets in GFP high cells (Adapted from Lim et. al)
Top 10 Pathways

Name	Size	Enrichment score (ES)	Normalized ES (NES)	Nominal p-value	FDR q-value
HALLMARK_INFLAMMATORY_RESPONSE	183	0.59538126	2.160031	0	0
HALLMARK_INTERFERON_ALPHA_RESPONSE	80	0.6511026	2.112234	0	5.78E-04
HALLMARK_XENOBIOTIC_METABOLISM	177	0.5716842	2.0786657	0	3.85E-04
HALLMARK_INTERFERON_GAMMA_RESPONSE	170	0.57464147	2.0677285	0	2.89E-04
HALLMARK_IL6_JAK_STAT3_SIGNALING	84	0.6287455	2.0538242	0	2.31E-04
HALLMARK_TNFA_SIGNALING_VIA_NFKB	178	0.5683673	2.0413365	0	1.93E-04
HALLMARK_COAGULATION	116	0.5588027	1.8982741	0	5.47E-04

F

Differentially expressed antigen presentation genes (Adapted from Lim et. al)

Gene	B2m	H2-Aa	H2-Ab1	H2-DMa	H2-DMb2	H2-Eb1	H2-Q10	H2-Q4	H2-Q5	H2-T10	Tap1	Tap2	Tapbp	Psmb8	Psmb9
log2FC (GFPhigh/GFPneg)	0.482	4.047	4.251	1.808	1.291	2.059	0.532	1.189	1.562	0.822	0.91	0.795	0.985	0.816	0.987

Figure S15: Enrichment of inflammatory and antigen presentation genes in low-NE SCLC cell lines and a SCLC mouse model. (A) Segregation of SCLC cell lines into NE (n=59) and low-NE (n=7) phenotypes. **(B)** ssGSEA antigen processing machinery scores in NE and low-NE SCLC cell lines. **(C)** Correlation between ssGSEA antigen processing machinery and NE scores. 95% confidence interval for the line shown in grey shading. **(D)** Significant GSEA enrichment plots comparing low-NE to NE SCLC cell lines. **(E)** Top 10 hallmark pathways enriched in GFP high cells in a mouse model of SCLC. **(F)** Differentially expressed antigen presentation genes in GFP high vs. low cells in a $p53^{flox/flox}$;$Rb^{flox/flox}$;$p130^{flox/flox}$ conditional triple knockout (TKO) mouse model of SCLC. Statistical significance was tested with a two-tailed Mann–Whitney U-test. Boxplots represent the median, 25th and 75th percentiles and the vertical bars span the 5th to the 95th percentiles. Source data are provided as a Source Data file.
