# Supplementary material for: Notch signaling and efficacy of PD-1/PD-L1 blockade in relapsed small cell lung cancer
Source: Nat Commun. 2021 Jun 23;12:3880. doi: 10.1038/s41467-021-24164-y (PMC8222224; doi:10.1038/s41467-021-24164-y)
Supplement: Supplementary file 2 — Description of Additional Supplementary Files [file 41467_2021_24164_MOESM2_ESM.pdf]

Description of Additional Supplementary Files

Supplementary Data 1-7:
Description: NCI discovery cohort clinical and genomic data

Supplementary Data 8:
Description: TCR sequencing data

Supplementary Data 9-13:
Description: Rochester validation cohort clinical and genomic data

Supplementary Data 14:
Description: Combined NCI discovery and Rochester validation cohorts

Supplementary Data 15:
Description: Moffit Cohort Clinical Data

Supplementary Data 16:
Description: Combined SCLC RNA-seq Datasets

Supplementary Data 17:
Description: SCLC cell lines microarray data

Supplementary Data 18:
Description: Pathways and genes used for ssGSEA analyses

Supplementary Data 19:
Description: Primer Sequences
