# Supplementary material for: Notch signaling and efficacy of PD-1/PD-L1 blockade in relapsed small cell lung cancer
Source: Nat Commun. 2021 Jun 23;12:3880. doi: 10.1038/s41467-021-24164-y (PMC8222224; doi:10.1038/s41467-021-24164-y)
Supplement: Supplementary file 12 — Reporting Summary [file 41467_2021_24164_MOESM12_ESM.pdf]

nature research

Corresponding author(s): Anish Thomas

Last updated by author(s): 05/21/2021

Reporting Summary

Nature Research wishes to improve the reproducibility of the work that we publish. This form provides structure for consistency and transparency in reporting. For further information on Nature Research policies, see our Editorial Policies and the Editorial Policy Checklist.

Statistics

For all statistical analyses, confirm that the following items are present in the figure legend, table legend, main text, or Methods section.

n/a	Confirmed	
☐	☒	The exact sample size (n) for each experimental group/condition, given as a discrete number and unit of measurement
☐	☒	A statement on whether measurements were taken from distinct samples or whether the same sample was measured repeatedly
☐	☒	The statistical test(s) used AND whether they are one- or two-sided<br>*Only common tests should be described solely by name; describe more complex techniques in the Methods section.*
☐	☒	A description of all covariates tested
☐	☒	A description of any assumptions or corrections, such as tests of normality and adjustment for multiple comparisons
☐	☒	A full description of the statistical parameters including central tendency (e.g. means) or other basic estimates (e.g. regression coefficient) AND variation (e.g. standard deviation) or associated estimates of uncertainty (e.g. confidence intervals)
☐	☒	For null hypothesis testing, the test statistic (e.g. F, t, r) with confidence intervals, effect sizes, degrees of freedom and P value noted<br>*Give P values as exact values whenever suitable.*
☒	☐	For Bayesian analysis, information on the choice of priors and Markov chain Monte Carlo settings
☒	☐	For hierarchical and complex designs, identification of the appropriate level for tests and full reporting of outcomes
☐	☒	Estimates of effect sizes (e.g. Cohen's d, Pearson's r), indicating how they were calculated

Our web collection on statistics for biologists contains articles on many of the points above.

Software and code

Policy information about availability of computer code

Data collection	No custom code was used.
Data analysis	BWA, DESeq2, VDJTools, Gene Pattern, RSEM, ClinOmics Somatic Bioinformatic pipeline and R-studio software (versioon 1.4.1106) was used for data analyses.

For manuscripts utilizing custom algorithms or software that are central to the research but not yet described in published literature, software must be made available to editors and reviewers. We strongly encourage code deposition in a community repository (e.g. GitHub). See the Nature Research guidelines for submitting code & software for further information.

Data

Policy information about availability of data

All manuscripts must include a data availability statement. This statement should provide the following information, where applicable:

- Accession codes, unique identifiers, or web links for publicly available datasets
- A list of figures that have associated raw data
- A description of any restrictions on data availability

The raw exome and RNA-seqeuncing data generated in this study have been deposited in dbGaP under accession code phs002176.v1.p1 [https://www.ncbi.nlm.nih.gov/projects/gap/cgi-bin/study.cgi?study_id=phs002176.v1.p1]. Raw TCR sequencing data is available at: https://github.com/nitinroper/SCLC-ICB-NCI. Additional RNA-sequencing datasets used in this manuscript are available in dbGaP under accession number phs001049.v1.p1 [https://www.ncbi.nlm.nih.gov/projects/gap/cgi-bin/study.cgi?study_id=phs001049.v1.p1], Gene Expression Omnibus under accession numbers GSE60052 [http://www.ncbi.nlm.nih.gov/geo/query/acc.cgi?acc=GSE60052] and GSE43346 [https://www.ncbi.nlm.nih.gov/geo/query/acc.cgi?acc=GSE43346], European Genome-Phenome Archive under accession number EGAS00001000925 [https://ega-archive.org/studies/EGAS00001000925]. The processed data are available in the Supplementary Data. Source data are available as a Source Data file. The remaining data are available within the Article, Supplementary Information or available

from the authors upon request (Nitin Roper, nitin.roper@nih.gov or Anish Thomas, anish.thomas@nih.gov).

Field-specific reporting

Please select the one below that is the best fit for your research. If you are not sure, read the appropriate sections before making your selection.

☒ Life sciences ☐ Behavioural & social sciences ☐ Ecological, evolutionary & environmental sciences

For a reference copy of the document with all sections, see nature.com/documents/nr-reporting-summary-flat.pdf

Life sciences study design

All studies must disclose on these points even when the disclosure is negative.

Sample size	The trial was conducted with use of an optimal two-stage phase II trial design to rule out an unacceptably low ORR rate of 15% in favor of an improved response rate of 35% with an alpha value of 0.10 and beta value of 0.10. Futility was defined as zero to three responses in the first 19 patients; accrual would continue to 33 patients if there were four or more responses in the first stage. The trial enrolled 20 patients, but closed to further accrual after only 2 of 19 patients had responses (Ref 15)
Data exclusions	All samples with insufficient tumor for RNA-sequencing were excluded from analyses. In discovery cohort, one sample with post-pathology review of atypical carcinoid was excluded and in validation cohort a sample was later determined to be from a patient with a non-small cell lung cancer diagnosis therefore this patient was excluded.
Replication	There were no attempts for replication of Figures 1-4 due to the nature of the study. At least three replicates were used for experimental studies shown in Figure 5.
Randomization	There were no randomizations as the original study was a single-arm clinical trial.
Blinding	Investigators who were responsible for obtaining samples for the validation cohort were blinded to the discovery cohort results. Additionally, investigators who performed any immunohistochemistry scoring were also blinded to the cohort results.

Reporting for specific materials, systems and methods

We require information from authors about some types of materials, experimental systems and methods used in many studies. Here, indicate whether each material, system or method listed is relevant to your study. If you are not sure if a list item applies to your research, read the appropriate section before selecting a response.

Materials & experimental systems

n/a	Involved in the study	
☐	☒	Antibodies
☐	☒	Eukaryotic cell lines
☒	☐	Palaeontology and archaeology
☒	☐	Animals and other organisms
☐	☒	Human research participants
☐	☒	Clinical data
☒	☐	Dual use research of concern

Methods

n/a	Involved in the study	
☒	☐	ChIP-seq
☒	☐	Flow cytometry
☒	☐	MRI-based neuroimaging

Antibodies

Antibodies used	The antibodies used were cleaved NOTCH1 (CST 4147; dilution 1:1000), HES1 (CST 11988; dilution 1:1000), YAP1(CST 4912; dilution 1:1000), MHC Class I (Hokudo AB-46-H; dilution 1:10000), PSMB8 (CST 13635; dilution 1:1000), β2M (CST 12851
Validation	All antibodies are well-validated by manufacturers and are highly cited within the literature

Eukaryotic cell lines

Policy information about cell lines

Cell line source(s)	ATCC NCI-82
Authentication	STR testing
Mycoplasma contamination	Cell lines were routinely tested for mycoplasma and were negative prior to each experiment.

Commonly misidentified lines (See ICLAC register)	No commonly misidentified lines were used in this study.

Human research participants

Policy information about studies involving human research participants

Population characteristics	Full population characteristics are listed in Supplementary Tables 1, 9 and 15
Recruitment	Please refer to Reference #15
Ethics oversight	NCI, University of Rochester and Moffitt Cancer Center IRB approved

Note that full information on the approval of the study protocol must also be provided in the manuscript.

Clinical data

Policy information about clinical studies

All manuscripts should comply with the ICMJEguidelines for publication of clinical research and a completedCONSORT checklist must be included with all submissions.

Clinical trial registration	NCT02484404 (https://www.clinicaltrials.gov/ct2/show/NCT02484404)
Study protocol	Full clinical protocol for discovery cohort is available upon request.
Data collection	Please refer to Reference #15
Outcomes	Please refer to Reference #15
